# Comparative multi-goal tradeoffs in systems engineering of microbial metabolism

**DOI:** 10.1186/1752-0509-6-127

**Published:** 2012-09-26

**Authors:** David Byrne, Alexandra Dumitriu, Daniel Segrè

**Affiliations:** 1 Bioinformatics Program, Boston University, Boston, MA, 02215, USA; 2 Department of Biology, Boston University, Boston, MA, 02215, USA; 3 Department of Biomedical Engineering, Boston University, Boston, MA, 02215, USA

**Keywords:** Metabolism, Microorganisms, Metabolic engineering, Constraint-based modeling

## Abstract

**Background:**

Metabolic engineering design methodology has evolved from using pathway-centric, random and empirical-based methods to using systems-wide, rational and integrated computational and experimental approaches. Persistent during these advances has been the desire to develop design strategies that address multiple simultaneous engineering goals, such as maximizing productivity, while minimizing raw material costs.

**Results:**

Here, we use constraint-based modeling to systematically design multiple combinations of medium compositions and gene-deletion strains for three microorganisms (*Escherichia coli*, *Saccharomyces cerevisiae*, and *Shewanella oneidensis*) and six industrially important byproducts (acetate, D-lactate, hydrogen, ethanol, formate, and succinate). We evaluated over 435 million simulated conditions and 36 engineering metabolic traits, including product rates, costs, yields and purity.

**Conclusions:**

The resulting metabolic phenotypes can be classified into dominant clusters (meta-phenotypes) for each organism. These meta-phenotypes illustrate global phenotypic variation and sensitivities, trade-offs associated with multiple engineering goals, and fundamental differences in organism-specific capabilities. Given the increasing number of sequenced genomes and corresponding stoichiometric models, we envisage that the proposed strategy could be extended to address a growing range of biological questions and engineering applications.

## Background

Microorganisms possess metabolic capabilities that are essential to society, science, and industry. Today, most bulk and specialty chemicals are derived from crude oil. However, declining oil reserves, rising oil prices, and growing environmental concerns have prompted renewed interest in producing chemicals using microorganisms instead of fossil fuels [1]. To transform microbial hosts into cellular factories, the applied discipline of systems metabolic engineering is using genome-scale approaches that redirect microbial metabolism to synthesize renewable and cost-effective biochemicals [1-3].

Classical metabolic engineering methods use localized metabolic intuition and random mutagenesis screening to develop microbial strains that possess improved biochemical production capabilities. For example, *Escherichia coli* does not naturally produce succinic acid as a major fermentative product; consequently, early metabolic engineering efforts targeted metabolic pathways that were thought to be involved in succinic acid synthesis. However, these perceived improvements were often ineffective or produced undesirable side-effects (e.g. large amounts of impurities were produced or cell growth was significantly inhibited) [4-7]. While some conventional strategies have shown a degree of success, production levels for the synthesis of succinic acid, as well as many other valuable biochemical compounds, often fall considerably short of maximum theoretical production limits [1,3,8]. These shortcomings are due, in part, to the fact that metabolic pathways and related regulatory processes form complex molecular and functional interaction networks. By focusing solely on one particular enzyme or metabolic pathway, it is likely that interrelated and potentially undesirable effects elsewhere in the cell might be inadvertently missed [9].

Similarly, conventional metabolic designs often singly focus on achieving maximal production rates or yields of targeted compounds without accounting for adverse economic consequences (e.g., due to material costs or final product purification processes) that may ultimately make a design impractical or commercially infeasible. Many, or even most, real engineering problems have multiple engineering goals, such as maximizing operational performance, minimizing material cost, and maximizing experimental reproducibility. The criteria subsequently used for design and optimization of engineering processes largely depend on which engineering goals are chosen. For industrial fermentation processes, four of the most important design-selection criteria are productivity, yield, final titer, and economic cost [10]. Productivity is the rate of product generation and is important to ensure the effective utilization of production capacity (e.g. capacity of bioreactors). Yield is the ratio of unit product formation to unit substrate consumption and is used as a measure of the production efficiency. Final titer is the purity of product generation and is important since further treatment of the fermentation medium, such as removal of impurities, may be necessary. Finally, economic cost is the monetary expenditure per unit of generated product. Economic costs may be associated with each component of the fermentation process and may ultimately dictate the viability of a product given current market conditions. Furthermore, engineering criteria may be condition-dependent (e.g. the criteria used for high volume, low value-added industrial fermentation products may differ significantly from the criteria used for low volume, high value-added products) and conflicting (e.g. the goal of maximum productivity may adversely affect the goal for minimum economic cost). Thus, tradeoffs among engineering goals can help to differentiate and prioritize design selection criteria.

To help evaluate and understand these complex biological and engineering relationships, system modeling is becoming an increasingly valuable tool for scientists and metabolic engineers alike. Kinetic modeling has been used to evaluate dynamic enzymatic effects of metabolism [11]. However, at whole-cell scales, kinetic modeling can become unwieldy due, in part, to the prerequisite of kinetic parameters that may be difficult to obtain experimentally [12]. Consequently, constraint-based modeling has become a powerful alternative, since it obviates this prerequisite by approximating metabolism in steady-state [9]. Despite this simplification and some additional limitations [12], constraint-based modeling has been experimentally shown to provide valuable predictions of whole-cell metabolic fluxes and growth phenotypes under a variety of environmental and genetic conditions [13-15]. As a result, a growing number of constraint-based analysis methods are being developed to evaluate metabolic models and the corresponding mathematical solution space that characterizes the phenotypic potential of an organism [9]. For example, flux balance analysis (FBA) uses a chosen objective function to search the edges of the mathematical solution space for a single optimal network state and associated flux distribution [16]. FBA has been used for a variety of applications, such as predicting the lethality of gene knockouts [17] and quantitatively predicting cellular growth rates and fluxes under different conditions [14]. Bi-level optimization approaches based on FBA [18] have been developed to simultaneously optimize two hierarchically-related objectives such as a primary and secondary metabolite production in microbial strain design. In particular, an initial algorithm aimed at identifying optimal designs through multiple gene knockouts (OptKnock [19]) was followed by more versatile approaches capable of taking into account gene up-regulation and down-regulation (OptReg [20]), as well as existing flux measurements (OptForce [21]). Rather than analyzing single network states, other constraint-based analysis techniques, such as extreme pathway analysis [22] and uniform random sampling [23], may be used to assess global network properties, characterizing ranges of optimal or sub-optimal biochemical network states. In addition, to address multiple optimality goals that may conflict and cannot be optimized simultaneously, multi-objective optimization and trade-off analysis approaches have been recently developed [24-26]. Together, these methods are yielding new biological and engineering insights.

In this study, we develop an integrative computational framework that elucidates relationships between environmental and genetic perturbations and their system-wide effects on microbial metabolism and metabolic engineering design strategies. Prior metabolic engineering studies have primarily focused on either environmental or genetic perturbation strategies, a single organism, one or a few engineering goals (usually productivity or yield) and optimal design solutions. Conversely, our approach addresses the multifaceted nature of metabolic engineering design processes by exhaustively generating and systematically analyzing more than four hundred million designs that incorporate both extracellular (i.e. medium composition) and intracellular (i.e. genetic knockout) perturbations and multiple microorganisms and engineering goals. Although any biochemical reaction network and synthesized target metabolite can be incorporated into our methodology, we focus on three microorganisms (*E. coli*, *S. cerevisiae* and *S. oneidensis*) and six target metabolite by-products of industrial interest: acetate [27], ethanol [28,29], formate [30], hydrogen [31], D-lactate [27,32], and succinate [33-35]. *Escherichia coli*[13,36] and *Saccharomyces cerevisiae*[35] are perhaps the best characterized and studied prokaryotic and eukaryotic microorganisms, respectively, and are commonly used for a wide range of computational and experimental scientific studies and industrial applications. *Shewanella oneidensis*[37] is, by comparison, a more recently sequenced [38] and less well-studied bacterium, yet it possesses considerable potential for bioremediation [37], microbial fuel cells [39] and other bioenergy applications [31]. A set of 36 biological and economic traits is used to evaluate corresponding engineering design goals. Although economic considerations are paramount in evaluating feasibility of any industrial design with commercial potential, a methodology for incorporating economic factors into constraint-based modeling had not been implemented before. The resulting population of phenotypes provides a rich dataset that is used to assess local design considerations and biological causalities, as well as global perturbation effects. An experimental compatibility score is used to assess the expected agreement of predictions with experimental data, such as mRNA expression arrays. Additionally, we present local tradeoffs between individual designs and engineering goals and global tradeoffs of metabolic traits across and within organisms. We find distinctive phenotypic characteristics that differentiate innate organism-specific metabolic capabilities, making certain organisms more suitable for particular engineering applications. We also find specific and general metabolic design strategies that can be used to facilitate optimal engineering output.

## Results

### Generation of engineering design candidates

**Figure 1:**
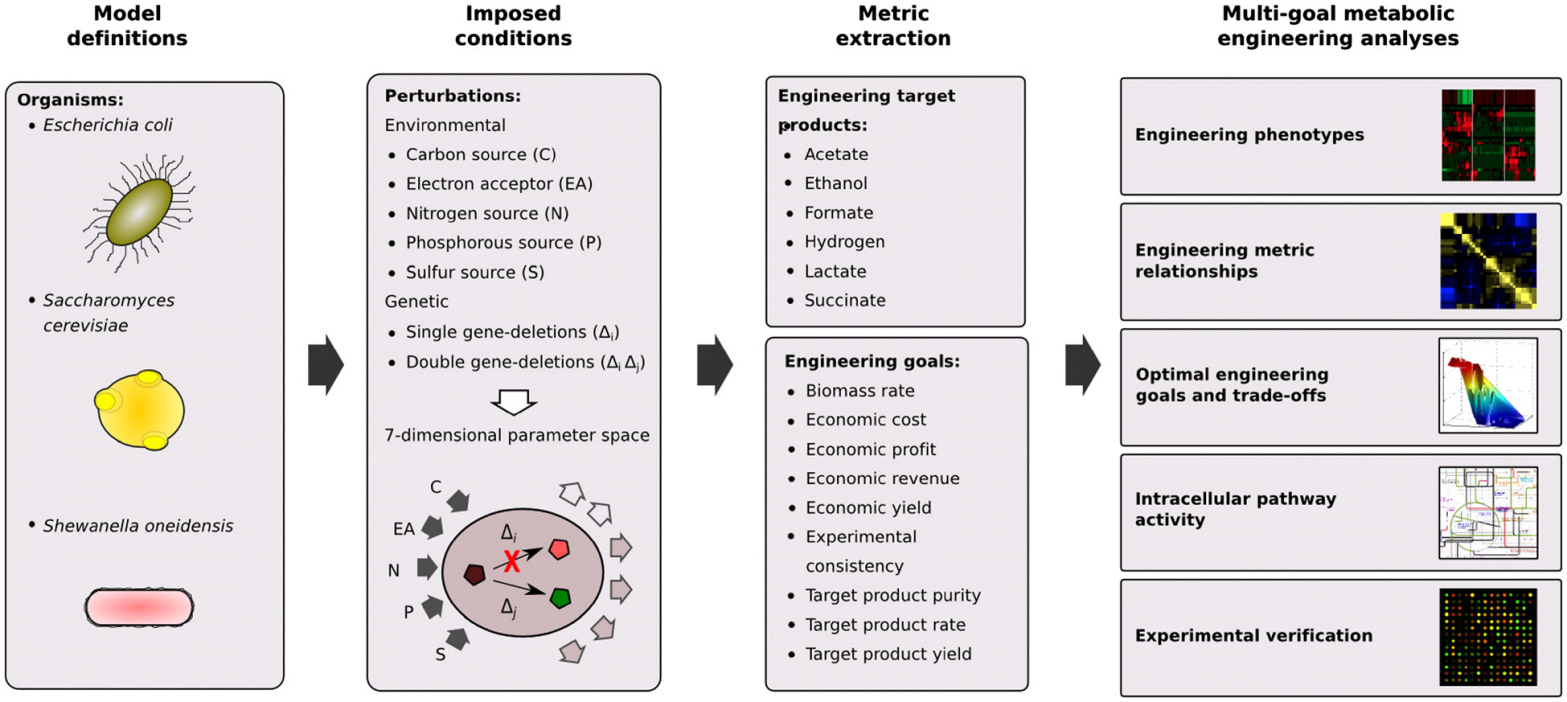
**Procedural overview.** The multi-goal metabolic engineering process incorporates three main decision-variable components: (1) organism model definitions, (2) imposed environmental and genetic conditions, and (3) extraction of desirable engineering metrics and goals. The resultant extracellular engineering phenotypes, relationships between the decision variables, and intracellular pathway activity can then be analyzed and experimentally verified to provide mechanistic insight and achieve optimal engineering designs.

As a first step we sought to generate a large computational dataset and a statistical analysis pipeline from which we could identify optimal strategies for the production of different molecular compounds. Our predictions, based on flux balance analysis, span different organisms, experimental design schemes and output metrics. Our computational approach (Figure 1) incorporates three main decision variable components: organism model definitions, imposed environmental and genetic conditions, and a set of engineering metrics for extraction. Additional file 1: Table S1 lists the model attributes and perturbations associated with the three genome-scale metabolic models used in this study: *E. coli*, *S. cerevisiae*, and *S. oneidensis*. Perturbations are categorized by nutrient type (carbon, electron acceptor, nitrogen, phosphorous, and sulfur sources) and single or double gene deletions. An engineering *design* consists of a specific organism, feedstock composition and genotype (e.g. *E. coli* Δedd Δgnd mutant grown anaerobically on glucose, ammonium, phosphate, and sulfur minimal medium). Combinations of organisms and perturbation parameters are exhaustively enumerated to simulate different conditions, producing a population of candidate designs. A set of generic metabolic *traits* (Additional file 1: Table S2) is defined to characterize the engineering designs and the phenotypic states of the metabolic system (e.g. target-compound carbon yield is a measure of carbon usage efficiency). The metabolic traits are functions of specific targeted-compound secretion rates, economic cost rates associated with the consumed media, or other measurements of metabolic activity (e.g. formate carbon yield is the proportion of the carbon consumed and utilized specifically for formate production). Engineering *goals* are particular metabolic traits that are preferentially either maximized or minimized to achieve a desired outcome (e.g., maximize formate carbon yield). The search for designs that are closest to the desired engineering goals is performed in two main steps: First, we solve an FBA problem for each combination of nutrients and gene deletions, using maximization of growth (biomass production) as the objective function. During this first step we prune out all designs that are non-viable. This constitutes a significant fraction of the designs tested (88%), but it still leaves a large number of viable solutions (~5 × 10^7^) to choose from. Second, we analyze all viable solutions found, and search for designs that optimize the engineering objective(s). During this stage of the optimization, we do not conduct any additional FBA, but rather perform a complex search among the previously computed designs. This also implies that once a design has been computed, the different phenotypes don’t have to be re-computed, but are just extracted from the data. We would like also to stress that, while in our case the two optimization steps are performed in two distinct procedures, the philosophy is similar to previous methods that perform the two steps in a single optimization algorithm (such as OptKnock [19]).

In total, more than 435 million conditions were simulated: 133,420,920 for *E. coli*, 179,133,985 for *S. cerevisiae*, and 123,124,374 for *S. oneidensis*. A relatively small fraction of these conditions produce viable-growth phenotypes (Additional file 1: Figure S1): 15% for *E. coli*, 11% for *S. cerevisiae*, and 9% for *S. oneidensis*. Thirty-six metabolic metrics (18 of which are functions of economic variables) are computed for each viable-growth phenotype. Box-plot statistics for the complete data set are shown in Additional file 1: Figure S2. Economic data were available for 80% of *E. coli* nutrients, 71% of *S. cerevisiae* nutrients, and 63% of *S. oneidensis* nutrients. Unless specified otherwise, subsequent analyses are performed on the economic data subset (see Methods for more details). Experimental data used for estimating an experimental consistency score were available for 149 of the simulated conditions. While in this work we use an indirect measure of experimental consistency and do not present a direct comparison of predicted and measured fluxes, we wish to emphasize that flux balance models have undergone a number of experimental tests [40-43], and have been used successfully for different specific metabolic engineering applications, such as production of lycopene and vanillin [40-43].

### Different organisms are better at achieving different goals

**Figure 2:**
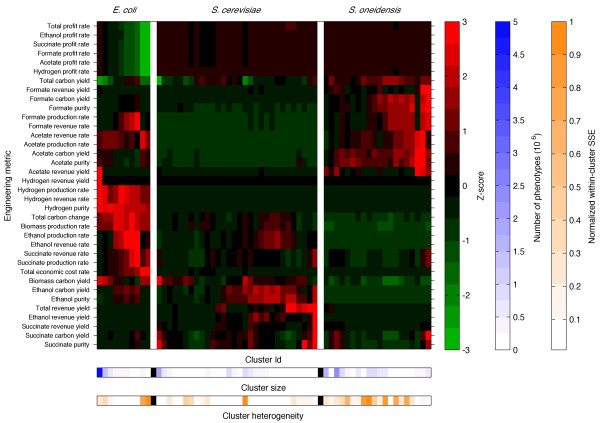
**Engineering meta-phenotypes for economic data set.** Each column (listed by Cluster Id and grouped by organism) is the centroid associated with a corresponding k-means phenotype cluster (meta-phenotype) for the simulation data subset with economic metrics. Cluster Ids increase sequentially, from left to right: 1 to 10 for *E. coli*, 11 to 30 for *S. cerevisiae*, and 41 to 60 for *S. oneidensis*. To compare across phenotype clusters and organisms, the metric values have been transformed into row-wise z-scores. All the rows and organism-specific columns were then hierarchically clustered. The “cluster sizes” are the number of individual phenotype simulations associated to each phenotype cluster. The “cluster heterogeneity” is the within-cluster sum of squared errors (SSE) for each phenotype cluster normalized by the maximum for each organism.

Given the enormous amount of data collected through the simulations across organisms and designs (approximately 6 terabytes of data in totality), it is a significant challenge to sort through and visualize this information in a useful manner. Using the metabolic traits, we classified the resultant viable phenotypes into dominant engineering meta-phenotypes for each organism. Classification was achieved by using iterative k-means clustering on the set of engineering phenotypes until an optimal cluster number was identified (Methods and Additional file 1: Figure S3). Each meta-phenotype vector is the computed centroid for the phenotypes within the associated cluster. Along with cluster sizes and heterogeneities, the meta-phenotypes succinctly describe the frequency and similarity of dominant phenotypic characteristics. For the economic dataset, we found 10, 30, and 20 meta-phenotypes for *E. coli*, *S. cerevisiae*, and *S. oneidensis*, respectively. To compare metabolic traits across phenotypes and organisms, the data are transformed into row-wise z-scores. The transformed data are then grouped by organism and hierarchically bi-clustered. The resultant “phenotypic maps” (Figure 2, Additional file 1: Figure S4 and Additional file 2: Table S4) provide a condensed global perspective of dominant phenotypic characteristics and metabolic capabilities that can quickly and easily be scanned to compare metabolic traits within and across organisms. A comprehensive list of all phenotype metrics and associated conditions may be downloaded or viewed using the online tool (see Methods).

Once organized into clustered meta-phenotypes, our data reveal that different organisms possess distinct dominant phenotypic characteristics. Figure 2 shows the meta-phenotypes for the subset of data with economic pricing. *E. coli* can be characterized by the fewest number of dominant meta-phenotypes (10) followed by *S. oneidensis* (20) and *S. cerevisiae* (30). The associated number of phenotypes is 10,086,971 for *E. coli*, 10,080,733 for *S. cerevisiae*, and 12,632,536 for *S. oneidensis*. The largest phenotype cluster for *E. coli* accounts for more than 56% of all phenotypes. That meta-phenotype has relatively low biomass production rates and high biomass carbon yields, as well as low profit rates due to lower rates of targeted byproducts synthesis. The second largest phenotype is similar to the first largest phenotype; the only major difference is considerably higher acetate and hydrogen revenue yields. Together, the two largest meta-phenotypes account for more than 70% of all phenotypes. This implies that for the metabolic traits and conditions under consideration, *E. coli* has relatively low phenotypic variation compared to *S. cerevisiae* and *S. oneidensis*, which are much more broadly distributed. The first two largest meta-phenotypes contain 30% and 36% of all phenotypes for *S. cerevisiae* and *S. oneidensis*, respectively. A comparison between the meta-phenotypes within and between organisms can be visualized in the form of a correlation matrix (Additional file 1: Figure S18).

Upon zooming into different regions of the meta-phenotype map (with representative engineering goals and conditions shown in Table 1 and Additional file 1: Tables S3 and S5), it is possible to identify specific meta-phenotypes that are associated with favorable strategies for a particular molecular product and goal. Figure 2 and Additional file 1: Figure S4 indicate that *E. coli* has a distinctively higher growth rate than *S. cerevisiae* and *S. oneidensis*, consistent with available experimental data [44]. *E. coli* readily produces ethanol, hydrogen, and acetate, whereas succinate and formate are produced only under very particular conditions. Comparatively, *S. cerevisiae* also readily produces ethanol (mainly yield and purity rather than production rate), whereas succinate is produced under particular conditions. *S. cerevisiae* produces little to no levels of hydrogen, acetate, and formate. *S. oneidensis* readily produces acetate and formate (mainly yield rather than purity), whereas hydrogen and ethanol are produced under fewer conditions. Since *E. coli* has a higher growth rate than *S. cerevisiae* and *S. oneidensis* (and therefore it would be expected to also have higher production rates), the higher yields and purities for formate and succinate for this organism are particularly noteworthy.

**Table 1:** Selected Pareto optimal engineering designs

**Design identifiers**		**Design criteria**		**Design annotations**			**Design metrics**						
**Design Id**^**a**^	**Cluster Id**^**b**^	**Design goals**^**c**^	**Design type**^**d**^	**Gene deletions**	**Nutrient sources**^**e**^	**Organism**^**f**^	**Acetate production rate (mmol gDW**^**-1**^ **hr**^**-1**^**)**	**Acetate purity**	**Biomass production rate (hr**^**-1**^**)**	**Microarray consistency**^**g**^	**Succinate production rate (mmol gDW**^**-1**^ **hr**^**-1**^**)**	**Succinate purity**	**Total economic cost rate ($ hr**^**-1**^**)**
1	8	Succinate production rate (0.99), Succinate purity (0.01)	candidate	edd, gnd	malthx, fum, gam, pi, so4	ec	0	0	1.85	NA	195.34	0.63	19314.4
2	8	Succinate production rate (0.5), Succinate purity (0.5)	candidate	atpH, caiD	malthx, fum, gam, pi, so4	ec	0	0	1.85	NA	181.57	0.71	19305.4
3	3	Succinate purity (1)	candidate	SO4417, SO3136	ac, fum, nh4, pi, so4	so	0	0	0.01	NA	20	1	3.72
4	1	Succinate production rate (0.01), Total economic cost rate (−0.99)	candidate	kgtP, lysP	sucr, o2, gam, ppt, so4	ec	36.17	0.35	1.85	NA	51.45	0.49	4.33
5	30	Succinate purity (0.5), Total economic cost rate (−0.5)	candidate	YBR196C, YMR256C	glc, o2, urea, pi, so4	sc	0	0	0.26	NA	14.77	0.43	0.05
6	1	Succinate production rate (0.33), Succinate purity (0.33), Total economic cost rate (−0.33)	candidate	SO4417, SO3136	glyclt, fum, nh4, pi, so4	so	0	0	0.06	NA	20	1	2.26
7	1	Succinate production rate (1)	validated	ptsG, pykFA, pfl	glc, NA, nh4, pi, so4	ec	4.28	0.24	0.12	NA	9.12	0.5	0.03
8	1	Acetate production rate (1)	microarray	appY	glc, NA, nh4, pi, so4	ec	6.76	0.24	0.17	0.46	0.06	0	0.75
9	1	Acetate purity (1)	microarray	arcA	glc, o2, nh4, pi, so4	ec	1.1	1	0.63	0.02	0	0	2.35
10	1	Microarray consistency (1)	microarray	arcA	glc, NA, nh4, pi, so4	ec	0	0	0.11	0.56	0.04	0	0.02
11	1	Acetate production rate (0.33), Microarray consistency (0.33), Acetate purity (0.33)	microarray	appY	glc, NA, nh4, pi, so4	ec	6.76	0.24	0.17	0.46	0.06	0	0.75

^a^ Engineering designs are referenced in the text by this identifier. ^b^ Each engineering design is associated with an engineering phenotype cluster (meta-phenotype) and identified according to the “Clusters” shown in Figure 6(A) for *E. coli*, Additional file 1: Figure S13(A) for *S. cerevisiae*, and Additional file 1: Figure S14(A) for *S. oneidensis*. ^c^ The specified engineering design goals and weights (shown in parentheses, where positive weights indicate goal maximization and negative weights indicate goal minimization) are used to determine the optimal engineering designs for the corresponding “Design type”. ^d^ “Validated” designs are designs that were experimentally validated, “microarray” designs are designs that had matching experimental microarray data, and “candidate” designs are simulated designs that are neither “validated” nor “microarray” designs. ^e^ Nutrients are ordered by nutrient type: carbon, electron acceptor, nitrogen, phosphorous, and sulfur source. “NA” indicates that no nutrient source of that type was provided. Metabolite abbreviations: ac = Acetate, fum = Fumarate, gam = D-Glucosamine, glc = D-Glucose, glu-L = L-Glutamate, glyclt = Glycolate, malthx = Maltohexoase, nh4 = Ammonium, o2 = O2, pi = Phosphate, ppt = Phosphonate, sbt-D = D-Sorbitol, so4 = Sulfate, sucr = Sucrose, urea = Urea. ^f^ Organism abbreviations: ec = *Escherichia coli*, sc = *Saccharomyces cerevisiae,* so = *Shewanella oneidensis*. ^g^ Engineering design simulations were compared with microarray data to compute experimental microarray consistency. “NA” indicates that no microarray data was available for the corresponding design.

It is apparent that different organisms are better at achieving different goals. *E. coli* tends to produce higher ethanol rates than the other organisms, whereas *S. cerevisiae* tends to produce higher ethanol purities and at better cost efficiency. Higher ethanol production rates in *E. coli* tend to be positively correlated with increased economic cost. *E. coli* also seems to be better at producing hydrogen, whereas *S. oneidensis* tends to be better at producing formate. Both *E. coli* and *S. oneidensis* are good at producing acetate. Under various conditions, all the organisms appear to be able to produce relatively high levels of succinate.

An additional outcome of this analysis is that economic considerations significantly affect optimal choices of engineering designs. This may be obvious, and commercial industries usually develop engineering strategies based on economic considerations. But, to our knowledge, this is the first time high-throughput FBA analysis has been combined with economic considerations. A very distinctive bimodal economic feature in the engineering phenotypic landscape (Figure 2) is that there are very expensive, high growth rate (with low profit rates and high ethanol production rates) designs and, conversely, there are cheaper and low growth rate designs. In *E. coli*, ethanol tends to be a costly product, whereas, by comparison, ethanol would seem to be more profitable in *S. cerevisiae*. In *E. coli*, acetate production tends to be more profitable than in the other two organisms.

### Pareto analyses and correlation maps reveal local and global trade-offs

**Figure 3:**
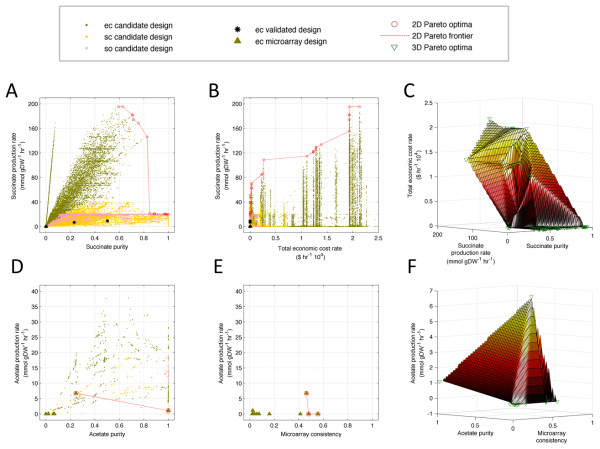
**Pareto optima and trade-offs for multiple engineering goals.(A-C)**. Two-dimensional (2D) and three-dimensional (3D) candidate and Pareto optimal designs and frontiers for engineering goals related to succinate production. Sub-optimal candidate designs for all organisms and experimentally validated *E. coli* designs are shown in **(A)** and **(B)**, but are not shown in **(C)** for clarity. **(D-F)**. 2D and 3D candidate and Pareto optimal designs that had matching experimental microarray data and frontiers for engineering goals related to acetate production. Error bars are too small to be visualized. For comparison, candidate designs that use the same media conditions as the microarray designs are shown in **(D)**, however only the microarray designs are used to compute Pareto optima and frontiers. Sub-optimal microarray designs are shown in **(D)** and **(E)**, but are not shown in **(F)** for clarity. The 3D Pareto frontiers shown in **(C)** and **(F)** are colored in proportion to the total economic cost and acetate production rates, respectively. Trade-offs can be determined by evaluating the rate of change of the Pareto frontier over the desired range of Pareto optimal designs. “Validated” designs are designs that were experimentally validated, “microarray” designs are designs that had matching experimental microarray data, and “candidate” designs are simulated designs that are neither “validated” nor “microarray” designs. Abbreviations: ec = *Escherichia coli*, sc = *Saccharomyces cerevisiae*, so = *Shewanella oneidensis*. The legend on top of the panels summarizes the symbols used.

The engineering goals currently being considered (Additional file 1: Table S2) can be optimized separately or in combination. If multiple goals conflict with each other, then one of the goals cannot be improved without simultaneously worsening at least one of the other goals. To address this problem we use Pareto analysis, which allows us to evaluate metric tradeoffs between designs and assist in the design selection process. While Pareto analysis can in principle yield solutions for any multidimensional trade-off, we focus here on two and three-dimensional cases, which can be readily visualized. Figure 3 and Additional file 1: Figure S5 show all candidate designs and Pareto optimal designs for multiple (two and three-dimensional) engineering goals.

While more detailed analyses for all six metabolic products are available in the Supplementary Materials and in the online tool (see Methods), we focus here, as a representative example, on all candidate designs relevant for the production of succinate in all three organism (Figure 3(A-C)). Results indicate that *E. coli* tends to have the greatest range of succinate capabilities, followed by *S. cerevisiae* and then *S. oneidensis*. The two-dimensional Pareto optimal frontier contains all multi-goal optimal designs and extends around the periphery of the solution space (Figure 3(A, B, D, E)). Piece-wise linear trade-offs are computed over a range of Pareto designs to determine the marginal gain or cost of relative changes in weighted linear combinations of goals. For example, for succinate production rate versus succinate purity (Figure 3(A)), there are 8 Pareto optimal designs within a range of succinate purity of 0 to 0.84 (succinate purity is defined as a ratio and is dimensionless; see Additional file 1: Table S2 for more details) and linear regression yields a tradeoff of −214.6 units of succinate production for every unit increase in succinate purity. Above succinate purity of 0.84, there are 16 Pareto optimal designs with tradeoff of −7.2 units of succinate production for every unit increase in succinate purity. This indicates that for unit increases in succinate purity below 0.84, there is a very large negative cost in succinate production rate, whereas above 0.84, where succinate purity is relatively high and succinate production rate is low, further increases in succinate purity come at relatively low additional cost in terms of succinate production rate decreases. Additionally, for succinate purity below 0.6 there are several *E. coli* Pareto optimal designs, between 0.6 and 1 there are many *S. cerevisiae* Pareto optimal designs, and for succinate purity close to 1 there is one *S. oneidensis* optimal design. Thus, *E. coli* is better for high succinate production rates and low succinate purity (with succinate purity sensitive to design changes), whereas *S. cerevisiae* (and to a small extent *S. oneidensis)* is better for high succinate purity and low succinate production rate (with comparatively low sensitivity to design changes). Similar logic can be applied to the Pareto optimal designs in three dimensions presented in Figure 3(B, C).

One may choose to prioritize simulated designs by their degree of consistency with available experimental data. High experimental consistency indicates that subsequent experimental validation may be more consistent with the predicted design solution. We computed experimental consistency scores by mapping available mRNA microarray data to metabolic flux values, in analogy with previously developed approaches to integrate gene expression data with FBA modeling (see Methods). Figure 3(E) shows designs considered for maximal acetate production rate, acetate purity and experimental consistency. Although designs with higher experimental consistency are preferable, Figure 3(E) indicates that higher microarray consistency comes at a cost of reduced acetate production rate. With additional higher resolution experimental data, such as metabolic flux measurements [45,46], these insights could be improved and expanded. In principle, prediction-mapped experimental data could be used as a proxy for predicting sensitivity or accuracy and as a metric for ranking designs.

The Pareto frontiers discussed above allow one to visualize different trade-offs identifiable from our data. It is further possible to focus on specific sections of these frontiers, and characterize engineering designs that are optimal for a specific linear combination of engineering goals. In general, we observe that different combinations of goals warrant very different design solutions. As illustrative examples, selected design criteria and associated optimal designs are presented in Table 1. Two of these designs yield maximal succinate production rate, both of which are for *E. coli*. Between the two designs, the design with higher succinate purity is an *E. coli* Δedd Δgnd mutant grown on minimal medium with maltohexoase as carbon source, fumarate as electron acceptor, D-glucosamine as nitrogen source, phosphate as phosphorous source, and sulfate as sulfur source. This design (hereafter referred to as Design 1, as specified in Table 1) produces 195.34 mmol succinate/gDW/hr with 0.63 succinate purity. Compared to Design 7 in Table 1 (an experimentally-validated succinate production design, *E. coli* ΔptsG ΔpykFA Δpfl mutant fermented on glucose minimal medium [8]), Design 1 has more than a 20-fold increase in succinate production rate. However, we also see that the total economic cost rate is very high (19314.4 $/hr), perhaps impractically so. Thus, we may alternatively choose design criteria that equally weight the maximization of succinate purity and minimization of total economic cost rate. Design 5 in Table 1 shows that this engineering goal combination produces a design for *S. cerevisiae* ΔYBR196C ΔYMR256C mutant aerobically grown on minimal medium with glucose as carbon source, urea as nitrogen source, phosphate as phosphorous source, and sulfate as sulfur source. Design 5 produces succinate purity of 0.43 and total economic cost rate of $0.05/hr. It also produces 14.77 mmol succinate/gDW/hr. As a result, this design has comparable economic cost to the validated *E. coli* ΔptsG ΔpykFA Δpfl mutant design (Design 7) [8], yet has 60% higher succinate production rate. In general, many of the resultant designs (including Design 1 and Design 5) do not appear in published literature and subsequent experimental validation of the simulation predictions will be warranted. It should be noted also that an effective implementation of Design 5 may be problematic, as it involves the deletion of a gene (YBR196C) previously reported to be essential under similar growth conditions, probably due to regulatory effects [47].

**Figure 4:**
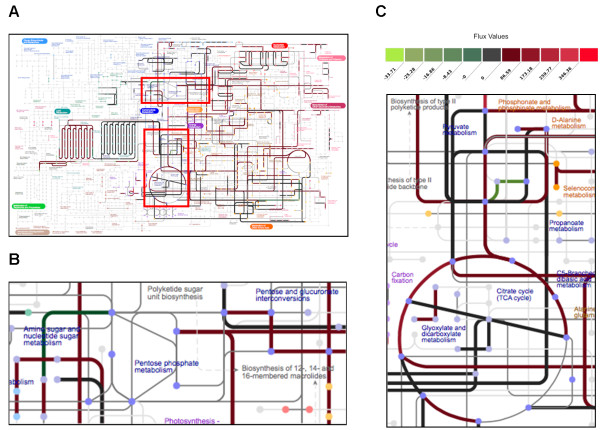
**Metabolic network pathway activity in *E. coli. *(A-C).** Differential pathway activity between the wild-type *E. coli* grown anaerobically on glucose minimal media and the mutant *E. coli* Δedd Δgnd grown anaerobically on maltohexoase, fumarate, D-glucosamine, phosphate, and sulfate minimal media (Design 1 in Table 1). **(A)** The complete metabolic network map, and close-ups (regions framed by red boxes) of **(B)** the citric acid cycle and **(C)** the lipid metabolism. Metabolic pathway reactions are color-coded according to the relative flux differences (color legend for flux values in **(C)** applies to **(A-C)**) between the engineering designs. Similar metabolic network maps for all organisms and Pareto optimal designs can be viewed using the Multi-Goal Metabolic Engineering Website (Methods).

In order to obtain more biological insight about the specific designs identified, we developed a new metabolic network visualization tool called Multi-Goal Metabolic Engineering (MGME) Visualizer that highlights the active fluxes for any given choice of engineering goals (Additional file 1: Figures S16 and S17 and online resource at http://nets.bu.edu). This interactive visualization of active fluxes makes it possible to identify potential causal connections between predicted phenotypes and underlying metabolic activity. Thus, MGME Visualizer constitutes a tool of broad practical applicability. Figure 4 shows the differential pathway activity results from the MGME Visualizer for Design 1 relative to a baseline design (wild-type *E. coli* grown fermentatively on glucose minimal medium, which are a typical laboratory strain and feedstock, respectively). We see that many of the reactions have relatively high flux values, which is expected since there is a large influx of carbon due to the use of maltohexoase (36 carbons per mole) in Design 1, compared to glucose (6 carbons per mole) in the reference design. Importantly, however, while the carbon input of Design 1 is 7 times larger than the carbon input of standard minimal medium, the predicted succinate rate increase is more than 2000–fold, demonstrating that the production improvement observed in Design 1 is largely a consequence of flux rerouting, rather than simply of additional carbon intake. In particular, from looking at the visualized flux differences, it is apparent that much of the flux is routing through the TCA cycle and is being channeled into the synthesis of succinate, a flux distribution trending towards maximum theoretical succinate production (Additional file 1: Figure S6(B)). The imposed gene-deletions Δedd and Δgnd amplify this effect since knocking out enzymes Entner-Douderoff dehydratase (EDD) and Gluconate-P dehydrogenase (GND) prohibits flux from being channeled into the pentose phosphate pathway and instead forces more flux into glycolysis, the TCA cycle, and finally succinate synthesis. A similar rerouting (though visually much more complex, due to intracellular compartments) can be observed for Design 5, based on yeast (Additional file 1: Figure S7).

**Figure 5:**
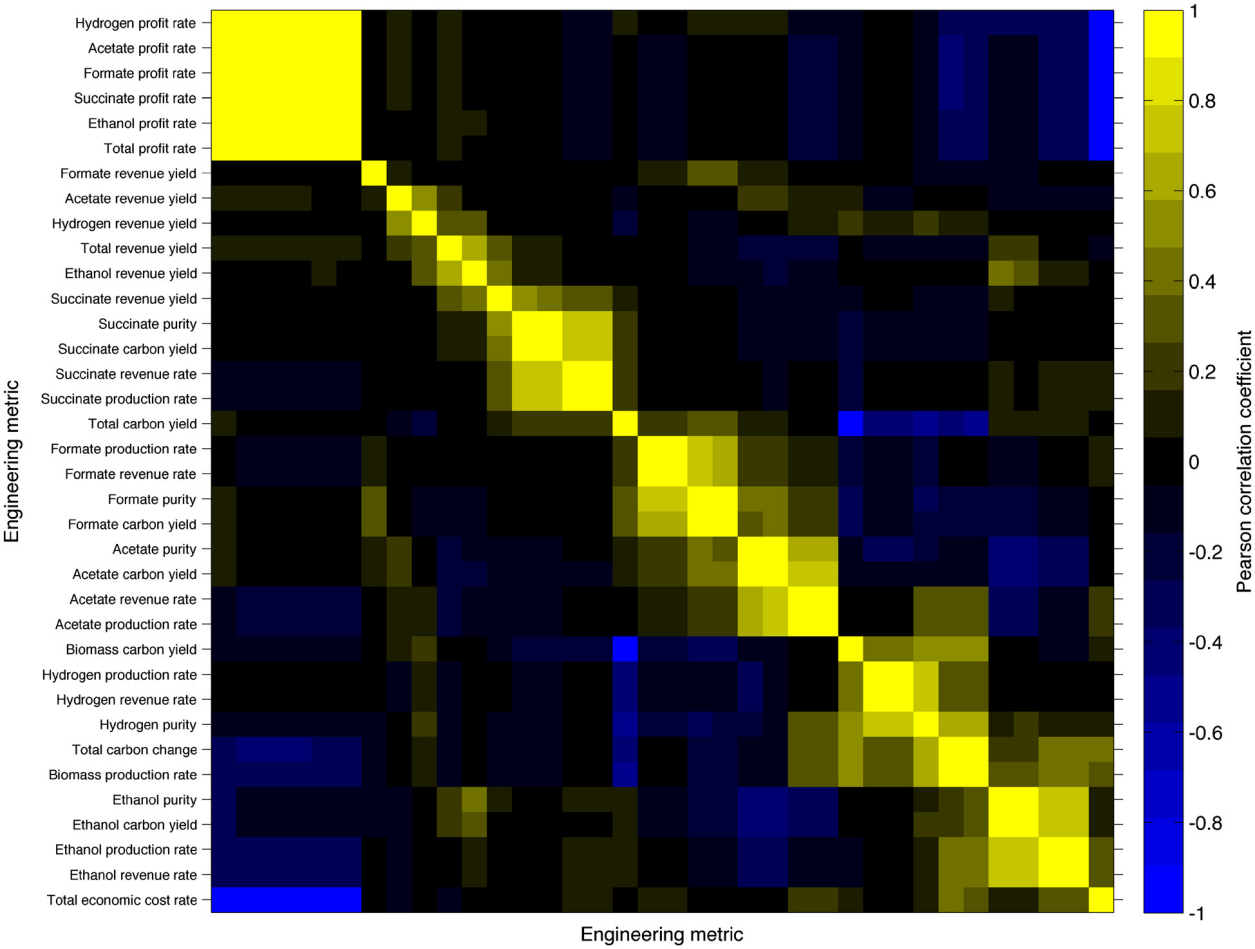
**Pair-wise correlations of engineering metrics.** Pearson correlations are computed between all pairs of engineering metrics for the combined organism data. Rows and columns are hierarchically clustered. Column labels are in the same order as the row labels, with the top metric row associated with the left-most metric column.

The above Pareto analysis focuses on the choice of an optimal design for a given set of desired goals. We reasoned that it would be interesting to obtain, in addition, a global snapshot of the tradeoffs between different metabolic traits. In fact, the large data set that we generated allows us to ask whether an increase of a given trait (e.g. a compound yield) is likely to be correlated or anti-correlated with another (e.g. the compound’s purity), and whether the observed tradeoffs are universal or organism-specific. We analyzed the data for all three organisms combined (Figure 5), and found that only a few general metabolic traits are highly correlated (5 pairs of engineering goals with |r| > 0.8). Biomass, as well as ethanol production rate, and total carbon change appear as correlated. Biomass production rate is highly anti-correlated with total overall carbon change and acetate production rate. Biomass carbon yield is highly anti-correlated with total carbon yield. Price change for all compounds (and for target compounds) is highly correlated with total intake price for all compounds. Profit rates tend to be anti-correlated with total economic cost rate and biomass production rate. By definition, profits and costs are inversely correlated. Additionally, economic profits are directly related to the over-production of desired products ethanol, hydrogen, formate, or succinate. This over-production is typically made possible by re-routing precursor metabolites away from biomass synthesis pathways and towards by-product synthesis pathways. As a result, the increased rates of targeted production tend to reduce the growth rate. In general, these conserved relationships tend to reinforce our biological intuition.

### Perturbation effects on engineering phenotypes are generalizable

So far, we focused on specific engineering goals. To provide an overall comparison of design strategies, we assessed the relative phenotypic effects of specific types of environmental and genetic perturbations. From a practical standpoint, a metabolic engineer would like to know what types of perturbations (i.e. gene deletions or environmental changes) are the most effective at inducing desirable phenotypes. To address this issue, a graph-based method was developed to assess the frequency at which different types of perturbations induce changes in the phenotypes. The availability of a huge number of phenotypic states provided the unique opportunity to explore the global connectivity between phenotypes. In particular, given any two meta-phenotypes, we asked how many elementary changes in nutrient conditions (e.g. carbon sources) or genetic background (e.g. single gene deletions) could mediate a transition between these two phenotypes. By computing the relative frequency at which a phenotype transitions from one meta-phenotype to another meta-phenotype, we can compare causal environmental and genetic perturbation types.

**Figure 6:**
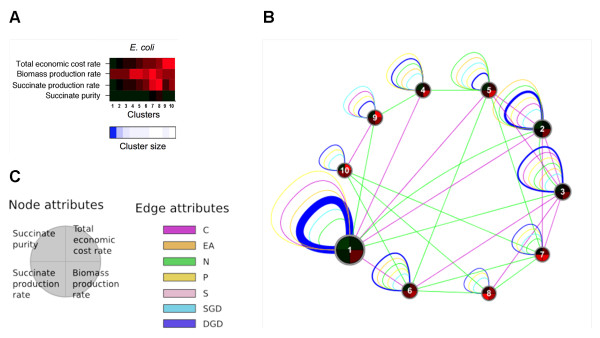
**Perturbation effects on phenotype changes in *E. coli*.(A)** A subset of the engineering metrics associated with *E. coli* phenotype clusters (meta-phenotypes) shown in Figure 2. For values of engineering metrics (z-scores) and cluster sizes, refer to legend in Figure 2. **(B)** Meta-phenotype transition network for *E. coli*. Nodes *i* and *j* represent two viable-growth engineering meta-phenotypes (the nonviable-growth meta-phenotype is not shown). Node labels correspond to Clusters shown in **(A)**. Node sizes are proportional to cluster sizes shown in **(A)**. Edge *t*_*i,j*_ represents the cumulative phenotype-cluster transition frequency between Nodes *i* and *j* due to a specified perturbation type. Edges are bidirectional, so *t*_*i,j*_ is equivalent to *t*_*j,i*_. Edge thickness is proportional to the cumulative transition frequency for environmental or genetic perturbations. Edges with relative frequency < 1% have been filtered out, primarily omitting low relative frequency single and double gene-deletion perturbations. **(C)** Legend for meta-phenotype transition network in **(B)**. Node faces are divided into quadrants that correspond to the selected engineering metrics shown in (A). Quadrant colors indicate the associated metric z-scores for the corresponding Cluster. Perturbation type (edge attribute) abbreviations: C = carbon sources, EA = electron acceptor sources, N = nitrogen sources, P = phosphorous sources, S = sulfur sources, SGD = single gene deletions, and DGD = double gene deletions.

Figure 6 and Additional file 1: Figures S13 and S14 show the resultant meta-phenotype transition networks for *E. coli*, *S. cerevisiae*, and *S. oneidensis*, respectively. Many interesting features are observed within and between these networks. Phenotypic variation can be evaluated based on the number of meta-phenotypes for each organism. For the 36 computed phenotypic traits, *S. cerevisiae* (30 meta-phenotypes) has the greatest phenotypic variation, followed by *S. oneidensis* (20 meta-phenotypes) and then *E. coli* (10 meta-phenotypes). This comparison implies that *S. cerevisiae* is the most versatile organism among the three, and can potentially serve a greater variety of metabolic engineering purposes associated with the six target metabolic products (acetate, ethanol, hydrogen, formate, succinate, and D-lactate).

Differences in the meta-phenotypic traits can be evaluated by comparing node colors. Each node face is divided into quadrants associated with the four selected engineering metrics: succinate production rate, succinate purity, biomass production rate, and total economic cost rate. For example, in Figure 6, meta-phenotype Nodes 9 and 10 are the most economically costly, whereas Nodes 7 and 8 have the highest succinate production rates. We comprehensively analyzed Design 1 and Design 5. Table 1 showed details of the designs and associated phenotype Cluster Ids. Design 1 is associated with Node 8 in Figure 6 and has high succinate production rate. Design 5 is associated with Node 30 in Additional file 1: Figure S13 and has high succinate purity.

Phenotypic prevalence is a measure of how common a meta-phenotype is, given the imposed conditions. Phenotypic prevalence is represented by node size, which is scaled by phenotype cluster size. We see that both Designs 1 and 5 are phenotypes associated with less prevalent meta-phenotypes. Node 1 in Figure 6 is the largest, and thus most common meta-phenotype for *E. coli*. We can similarly evaluate the distribution of phenotypic prevalence for each organism. The distribution for *E. coli* is the most skewed and, thus, the majority of *E. coli* phenotypes are associated with a few meta-phenotypes, whereas the distribution is comparatively more uniform for *S. cerevisiae* and *S. oneidensis*. Together with phenotypic variation, this further shows that for the studied traits *E. coli* has few overall dominant phenotypes and a single super-dominant meta-phenotype.

Some phenotypes are innately more robust or sensitive to different types of perturbations. Self-loops indicate the robustness of the phenotype cluster to genetic or environmental perturbations, whereas the thick edges between meta-phenotypes indicate that those phenotypes are sensitive to the considered type of perturbation. In Figure 6, phenotypes are generally robust against changes in electron acceptors but are relatively more sensitive to changes in carbon or nitrogen sources. Design 1 (associated with Node 8 in Figure 6) is relatively robust to changes in phosphorous sources, but more sensitive to carbon and nitrogen sources and single gene-deletions.

Network edges can be used to determine global transitions between meta-phenotypes. For example, Node 8 (meta-phenotype associated with Design 1) is connected to Nodes 6, 7, and 10 by nitrogen source perturbations and to Node 3 by carbon source perturbations. Thus, Node 8 is more closely related to those nodes and it is easier (i.e. fewer perturbations of those types are required) to transition between those nodes than between the other nodes that it is not connected to.

Interestingly, the different patterns of connectivity found in meta-phenotype graphs for different organisms suggest that, broadly speaking, the metabolic usefulness of different organisms may be best assessed through different types of perturbation analyses. Additional file 1: Figure S15 shows the relative perturbation influences on global phenotype changes. It is apparent that carbon and nitrogen source perturbations have the greatest relative effect on changes in phenotype in *E. coli*, whereas *S. cerevisiae*, and *S. oneidensis* are more uniformly sensitive to all types of environmental and genetic perturbations analyzed. Thus, if one is trying to perturb the *E. coli* metabolism, one might preferentially design carbon and nitrogen source perturbations.

## Discussion

We presented a high-throughput computational framework for generating and exploring an exhaustive landscape of *in silico* perturbations and metabolic engineering designs. Each design condition consists of an environment (medium composition), an organism (*Escherichia coli*, *Saccharomyces cerevisiae*, or *Shewanella oneidensis*) and a genotype (set of gene deletions). The vast population of the resultant design solutions produces a contextualized phenotypic map that is used to evaluate relationships between engineering goals and fundamental biological network properties. Using a set of metabolic traits, the large number of metabolic phenotypes is clustered into dominant meta-phenotypes. Whereas individual phenotypes are used to evaluate localized design considerations, causal biological mechanisms and design tradeoffs, the meta-phenotypes are used to evaluate global phenotypic diversity and relationships between metabolic traits and perturbation strategies. The proposed approach can help understand how environmental and genetic factors influence metabolism and metabolic engineering design.

A single unique optimal design solution may suffice for a single distinct set of weighted engineering goals. The resultant phenotypic map provides a regional and global context for this design solution relative to all other designs. If, for example, the exact values of the weights associated with the importance of the engineering goals are uncertain, we show that, by using the map, sub-optimal designs located in the proximity of optimality can be evaluated to assess the sensitivity of those weights. A designer can then assess whether or not it might be desirable to reprioritize engineering goals. By analogy, instead of having a single travel destination and navigational route to that destination, a map is very useful for assessing alternative destinations and routes that may, upon further inspection, be deemed more desirable than the original one.

Prior metabolic engineering studies have primarily focused on a single organism, engineering application, environment or genetic perturbation strategy, and optimal design solution. However, different organisms have different metabolic capabilities, due to diverse environmental adaptations and biochemical wiring. Thus, the interdependencies between the desirable organism, engineering application and design strategy are often unclear *a priori*. Here, we extended prior approaches by comparing organisms and systematically evaluating both environmental and genetic perturbation strategies. We illustrated inherent differences in the metabolic capabilities and phenotypic variations of *E. coli*, *S. cerevisiae* and *S. oneidensis*. To account for important economic considerations, we developed a methodology for integrating economic data. Furthermore, we showed that preexisting experimental data can be readily incorporated to help rank designs by how likely they are to be accurately reproduced experimentally.

After initial compilation of the phenotype population dataset, multiple complex combinatorial optimization problems can be solved (e.g. Pareto optimality design analysis). There is no restriction in terms of linearity or nonlinearity of metabolic traits and, if an engineering goal needs to be changed, there is no need to re-compute the phenotype dataset; one just needs to redefine the corresponding function and re-query the data. There are, however, limitations. For example, prediction accuracy of the metabolic designs is limited by the accuracy of the underlying models. Thus, accuracy can be further improved by improving the models (e.g. incorporating additional biological mechanisms, such as transcriptional regulation). Searching the design parameter space is also limited by the combinatorial nature of this “brute-force” approach. Here, we evaluated a comprehensive, but limited, subset of the theoretically infinite number of genetic and environmental parameter values and combinations. Simulating and processing the 435 million conditions took approximately 4 weeks of CPU time (see Methods for more details). By further optimizing the underlying programming code and by incorporating additional computing processors, the overall compute-time could be significantly reduced and many additional organisms and design strategies could be evaluated. Nevertheless, since an exhaustive search of the complete parameter space is not possible, prior knowledge will be useful in deciding which regions and level of granularity of the parameter space to explore.

Compared to other optimization approaches, our method potentially sacrifices depth (e.g. looking at triple and multiple knockouts) in favor of breadth (i.e. obtaining a snapshot of behaviors across an unprecedented number of perturbations and environments). Future studies may seek to further compare and contrast the spectrum of perturbation strategies to assess the advantages and limitations of each. For example, methods that can infer optimal combinations of more than two gene additions or deletions [19,41] could be preceded by broad surveys across multiple organisms. In addition, our approach could provide useful preliminary indication of the suitability of specific organisms for nonlinear objectives that may not be easily addressed through other available optimization approaches.

We would like to highlight that the biological details of our results can be conveniently accessed and visualized through the online tool that we present as part of this work. This tool is currently tied to predefined criteria for the choice of designs and engineering goals. However, future elaborations of our approach and of this tool could easily relax the existing constraints, for example including weights for the importance of different objectives, and thresholds for levels of acceptable violations of specific constraints. In addition, the process could be transformed into an iterative one, where an initial query throughout the entire space could be followed by a user-defined choice of specific criteria, which would lead to a deeper search in a restricted region of the space.

Furthermore, while in the current work we focus mainly on the metabolic phenotypes relevant for metabolic engineering applications, a different type of analysis could provide complementary insight on the biological aspects of the data presented. For example, it would be interesting to understand, for each meta-phenotype, whether it can be associated with specific environmental or genetic properties (e.g. limitation of a specific nutrient). This type of analysis would require revisiting our large data set (i.e. the meta-phenotypes shown in Additional file 2: Table S4, and the complete list of designs they comprise, available online, see Methods), in search for meaningful biological patterns.

## Conclusions

Given the increasing number of sequenced genomes, improved model accuracy and the growing available computing power, it is foreseeable that future extensions of our approach could help address a growing range of biological questions and engineering applications. Rapid growth of industrial biotechnology is helping to drive demand for a widening range of products, such as commodity chemicals (e.g., succinic acid and ethanol), fine chemicals (e.g. 6-aminopenicillanic acid and other antibiotics), and specialty chemicals (e.g., food and feed additives) [48]. In many application areas, however, production output of cellular factories falls significantly short of what is theoretically possible and may be insufficient for practical implementation. Systems engineering methods, including the approach presented, hold great promise in overcoming current engineering limitations and design challenges [1-3]. The exhaustive strategy we have explored, while combinatorially limited, enables complex searches across nonlinear objectives and multiple species, complementing other optimization methods, and providing a global portrait of the landscape of possible metabolic phenotypes.

## Methods

### Constraint-based modeling

The general equations describing the dynamics of a metabolic network can be written as

(1) $$\frac{d}{dt}C=Ax-\mu C$$

where **C** (mol/L) is the concentration vector of *m* internal metabolites, *x* (mol/L/h) is the reaction rate (flux) vector of *n* reactions, **A** is the stoichiometry matrix of dimension *m* × *n* whose elements *a*_*ij*_ represent the stoichiometric coefficient of the element *i* involved in reaction *j*, and *μ* (1/h) is the specific dilution rate associated with the change in volume of the system. At steady state there is no accumulation of internal metabolites in the system [49] and Equation (1) can be simplified to Ax = 0. Additionally, due to thermodynamic restrictions, some reactions can effectively be considered irreversible leading to additional contraints of the type *x*_*i*_ ≥ 0.

Flux balance analysis (FBA) is a method for predincting steady state reaction rates in a metabolic network [16]. Additional linear constraints are included to set upper and lower bounds on individual fluxes (*α*_*i*_ ≤ *x*_*i*_ ≤ *β*_*i*_) and are often used to impose maximal nutrient uptake rates. Upon choosing a linear objective function *f*(x) to be optimized, linear programming (LP) [50] is used to identify a solution subject to the constraints:

(2) $$\begin{array}{c} \max f\left(\text{x}\right)\\ s.t.\text{Ax}=0\\ \alpha_{i}\leq x_{i}\leq \beta_{i} \end{array}$$

An objective function that is commonly used for microbial systems is the maximization of biomass formation [51] (see Equation 3). To simulate changes in nutrient composition or gene-deletion effects over a range of parameter values, parameters *α*_*i*_ and *β*_*i*_ in the LP problem (Equation 2) can be iteratively modified (e.g., both set to zero to stimulate a gene knockout) and the problem solved again to obtain a new solution vector x. While this new solution achieves max *f*(x), the engineering objective (e.g. maximization of target-compound synthesis or minimization of media cost) may be suboptimal.

The genome-scale metabolic models for *E. coli*[52], *S. cerevisiae*[53], and *S. oneidensis*[44] are used to enumerate over a comprehensive set of feedstock medium compositions and single and double gene deletions. The nutrient and gene-deletion parameter space that is explored is described in Additional file 1: Table S1. Single and double gene-deletions are chosen from the genes associated with the citric acid cycle, glycolysis, gluconeogenesis, oxidative phosphorylation, pentose phosphate, and pyruvate metabolism pathways.

Medium nutrients are first categorized as carbon, electron acceptor, nitrogen, phosphate, or sulfur sources (progressively and exclusively, in that order) to enumerate all nutrient and gene-deletion combinations. The resultant candidate designs are then screened and selected to optimize for one or several specified engineering objectives. Upper bounds to nutrient uptake rates are computed based on the standard rates found in the literature [13,14,32,54]. Biomass production is incorporated as an additional reaction,

(3) $$\sum_{i}d_{i}z_{i}\to 1\;\text{biomass}$$

where the stoichiometric coefficient *d*_*i*_ corresponds to the experimentally measured contribution of biomass component *z*_*i*_ to biomass [51]. To quantify the engineering value of metabolic states under the various conditions, engineering metrics are defined as listed in Additional file 1: Table S2. Values for maximum theoretical engineering goals are computed using FBA (Equation (2)) using the engineering metrics themselves as objectives to be either maximized or minimized, rather than the objective function expressed in Equation (3). Linear programming is implemented using the GNU Linear Programming Kit software [55]. Data processing is implemented as a distributed process run on a computing cluster with 192 processor dual-dual core 2.8 Ghz computer nodes.

### Metabolic traits

Metabolic traits are defined in Additional file 1: Table S1. Final titer is typically measured as a concentration. However, it may alternatively be thought of as a ratio of the target product to the total by-products being produced (i.e. titer ratio).

Reagent prices were compiled from Sigma-Aldrich Corporation’s website http://www.sigmaaldrich.com/ on May 10, 2011. The nutrient unit prices ($/g) were computed using the largest reagent allotment size available and nutrient cost rates were computed as a product of the nutrient unit price, molecular weight, and flux:

$$\frac{\$}{g}\times \frac{g}{\text{mol}}\times \frac{\text{mmol}}{\text{hr}}\times \frac{\text{mol}}{1000\;\text{mmol}}$$

Unit prices for some nutrients were not available. Approximately 23% of the simulated medium compositions included one or more nutrients that did not have an assigned nutrient unit price. Additional file 1: Figures S2 and S4 show the results for all simulations. All other figures contain economic metric data based on nutrient unit prices and, therefore, omit those simulations with missing unit price data.

To evaluate how closely the simulated phenotype and pathway activity predictions correspond to the experimental values, a metric for experimental consistency is computed using a method called Gene Inactivity Moderated by Metabolism and Expression (GIMME) [56]. The GIMME algorithm provides a quantitative consistency score that indicates how consistent a set of gene expression data is when compared to a simulated flux solution under similar conditions. A set of 149 Affymetrix microarrays for *E. coli*, processed using GC-RMA [57], was gathered [56]. This method evaluates how closely the pathway activity, as measured by microarray gene expression, matches the simulated pathway flux activity.

### Meta-phenotypes

A combination of the computed metabolic traits can be considered a complex engineering phenotype. The engineering phenotypes across all the possible designs computed with FBA can be clustered into “meta-phenotypes” based on the similarity of their vectors. K-means clustering [58] was used with 10 seeds and up to 100 iterations to assign the engineering phenotypes into clusters that minimize the within-cluster average square-error:

$$E=\frac{1}{n}\sum_{i=1}^{k}\sum_{x\in C_{i}}{\left(x-m_{i}\right)}^{2}$$

where *m*_*i*_ = mean of cluster *C*_*i*_ and *n* = number of objectives in the dataset. To find the optimal number of clusters, the gap statistic [59] is used to compare within-cluster dispersions in the observed data to expected within-cluster dispersions in data generated from a null distribution when the deviation is maximized. This method, designed to be applicable to any cluster technique and distance measure, is in wide use [60-62]. We found that our optimal cluster numbers are fairly robust, particularly for the economic dataset discussed in the main text (Additional file 1: Figure S3B), where the deviation is significantly less for one cluster more or less than the computed optimal number of clusters.

To compare engineering metric values across meta-phenotypes, a z-score is computed as

$$\frac{\left(y_{i}-\bar{y}\right)}{\sigma }$$

where *y*_*i*_ is meta-phenotype *i*, $\bar{y}$ is the average meta-phenotype vector and σ is the meta-phenotype standard deviation. Instructions for downloading the phenotype metric data and conditions and associated meta-phenotypes mapping are available at http://nets.bu.edu/about.

### Pareto optimal designs and trade-offs

Multi-goal optimization (also known as multi-objective optimization) is the process of simultaneously optimizing two or more conflicting goals (or objectives) subject to a set of constraints [63-65]. In our study, we have a vector of engineering goals f(**v**) = *f*_1_(*v*),*f*_2_(*v*),…*f*_m_(*v*)], where *v* is the vector of computed engineering metrics shown in Table S2. The associated multi-goal optimization problem is min *f* (**v**), bounded by the discrete set of available solutions. Each solution corresponds to a metabolic engineering design candidate (or multiple candidates if they have identical engineering phenotypes). If the individual goals in *f (***v***)* do not conflict, then it is possible to find a unique optimal solution. However, if the individual goals in *f (***v***)* do conflict, then a unique solution will not exist. Instead, there will be a set of Pareto solutions. If a change (or tradeoff) in one of the solutions improves one goal without making another goal any worse, then that change is called a Pareto improvement and the initial solution is called dominated. If the subsequent solution is such that an improvement in one goal requires degradation in another goal, then that solution is called nondominated and is Pareto optimal. The set of Pareto optimal solutions is often called the Pareto frontier.

To determine the Pareto optimal designs and frontiers in our study, we use a Pareto-compliant method called Nondominated Sorting Genetic Algorithm II (NSGA-II) [66]. This method, widely used in prior research [25,67-69], incorporates a genetic algorithm and a ranking procedure to select nondominated solutions. Specifically, we used the Matlab function *gamultiobj*, from the Global Optimization Toolbox. We feed into the function our set of engineering goals, f(**v**), and obtain as a result the set of Pareto optimal designs. The parameter values that we used are 500 maximum number of generations, population size of 100 chromosomes, 0.85 probability of crossover, 0.05 probability of mutation, distribution index of 10 for simulated crossover, distribution index of 20 for simulated mutation and a random seed of 0.6. Prior studies showed that these parameter values are generally satisfactory [25,68] and we found that our results were not significantly sensitive to changes in these values. Subsequent Pareto tradeoff analysis (i.e. determining marginal gain or cost of relative changes in weighted linear combinations of goals) is computed using piece-wise linear differences between the Pareto designs associated with particular Pareto frontiers.

### Meta-phenotype transition network

Transition frequencies are computed by varying a single individual perturbation type (carbon, electron acceptor, nitrogen, phosphorous, and sulfur sources and single and double gene deletions), while maintaining fixed the remaining perturbation types. Environmental perturbations are imposed by changing the absence or presence of a nutrient in the medium (as described in the Methods “Constraint-based modeling” subsection), whereas genetic perturbations are imposed by deleting single or double genes. A resultant meta-phenotype transition network can be generated such that Nodes *i* and *j* represent two viable-growth engineering phenotype clusters. Edge *t*_*i,j*_ represents the cumulative phenotype-cluster transition frequency between Nodes *i* and *j* due to either environmental perturbations or genetic perturbations. Node *0* represents the nonviable-growth phenotype. Thus, edges *t*_*i,0*_ and *t*_*j,0*_ represent the cumulative phenotype-cluster transition frequencies to the nonviable-growth phenotype due to perturbations. Edges are bidirectional, so *t*_*i,j*_ is equivalent to *t*_*j,i*_. Edge thickness is proportional to the cumulative transition frequency for environmental or genetic perturbations. By performing this analysis systematically for all meta-phenotypes, we obtained a network of meta-phenotype transitions for each organism *E. coli*, *S. cerevisiae*, and *S. oneidensis*. Because the non-viable meta-phenotype is the sum of all possible non-viable phenotypes, it is comparatively extremely large and would effectively dwarf all viable meta-phenotype nodes. Thus, the non-viable meta-phenotype is not shown or included in the figures and results presented.

### Multi-goal Metabolic Engineering Visualizer

A public website (Additional file 1: Figures S16 and S17), located at http://nets.bu.edu, was developed to make available the optimal metabolic engineering design results. The website provides an interface that may be used to submit customized search queries, choose engineering designs, and interact with resultant metabolic network visualizations.

From the website’s main page (Additional file 1: Figure S16), a user can choose from a list of organisms, target products, and engineering goals. Based on selected optimization criteria, the website generates a list of metabolic engineering designs. If multiple engineering goals are selected, then a resultant set of Pareto optimal designs are tabulated where one can compare alternative designs with competing metric values. The user may click on any one of the designs to generate a metabolic network map that has corresponding metabolic pathways and reactions color-coded by flux values. The map can be panned, zoomed, and searched. Other map features include clickable nodes and edges for obtaining additional information about metabolites and reactions.

An online tutorial for the website (Additional file 1: Figure S17) is located at http://nets.bu.edu/about. Alternatively, the tutorial can be obtained by clicking “Help” on the website’s main page. The tutorial explains the process of defining engineering optimization criteria, selecting resultant designs and visualizing metabolic pathway activity.

## Abbreviations

ec/*E. coli*: Escherichia coli; EDD: Douderoff dehydratase; FBA: Flux balance analysis; GC-RMA: GC Robust Multi-array Average; gDW: Gram dry weight; GIMME: Gene Inactivity Moderated by Metabolism and Expression; GND: Gluconate-P dehydrogenase; LP: Linear programming; MGME: Multi-Goal Metabolic Engineering; sc/*S. cerevisiae*: Saccharomyces cerevisiae; so/*S. oneidensis*: Shewanella oneidensis; TCA: Tricarboxylic acid cycle.

## Competing interests

The authors declare that they have no conflict of interest.

## Authors’ contributions

DB conceived and designed the study. DB and AD generated the data and performed the statistical analyses. DB and DS interpreted the results and wrote the manuscript. DS supervised the project. All authors edited and approved the final version of the manuscript.

## Supplementary Material

Additional file 1 **Supplementary Information.** Tables S1, S2, S3, S5 and Figures S1-S18. Click here for file

Additional file 2 **Supplementary Information.** Table S4 (Tabulated version of Figure 2). Click here for file
