# Supplementary material for: Comparative multi-goal tradeoffs in systems engineering of microbial metabolism
Source: BMC Syst Biol. 2012 Sep 26;6:127. doi: 10.1186/1752-0509-6-127 (PMC3484036; doi:10.1186/1752-0509-6-127)
Supplement: Additional file 1 — Supplementary Information. Tables S1, S2, S3, S5 and Figures S1-S18. [file 1752-0509-6-127-S1.doc]

**Supplementary Information**

**Comparative multi-goal tradeoffs in systems engineering of microbial metabolism**

David Byrne^a,1^, Alexandra Dumitriu^a^ and Daniel Segrè^a,b^

^a^Graduate Program in Bioinformatics, Boston University, Boston, MA 02215, USA

^b^Department of Biology and Department of Biomedical Engineering, Boston University, Boston, MA 02215, USA

^1^ Corresponding author. E-mail: dbyrne@bu.edu, dsegre@ bu.edu

List of Figures

Supplementary Figure 1. Viable simulated conditions [3](#__RefHeading___Toc207085336)

Supplementary Figure 2. Box-plots of viable simulation results for complete data set [4](#__RefHeading___Toc207085337)

Supplementary Figure 3. Optimal phenotype cluster numbers [5](#__RefHeading___Toc207085338)

Supplementary Figure 4. Engineering phenotype clusters for complete data set [6](#__RefHeading___Toc207085339)

Supplementary Figure 5. Pareto optima for target-compound production rates [7](#__RefHeading___Toc207085340)

Supplementary Figure 6. Maximum theoretical succinate production in *E. coli* [8](#__RefHeading___Toc207085341)

Supplementary Figure 7. Metabolic network pathway activity in *S. cerevisiae* [9](#__RefHeading___Toc207085342)

Supplementary Figure 8. Pareto optima for total profit rate [10](#__RefHeading___Toc207085343)

Supplementary Figure 9. Pair-wise correlations of engineering metrics for *E. coli* [11](#__RefHeading___Toc207085344)

Supplementary Figure 10. Pair-wise correlations of engineering metrics for *S. cerevisiae* [12](#__RefHeading___Toc207085345)

Supplementary Figure 11. Pair-wise correlations of engineering metrics for *S. oneidensis* [13](#__RefHeading___Toc207085346)

Supplementary Figure 12. Pair-wise metric correlation comparisons between organisms [14](#__RefHeading___Toc207085347)

Supplementary Figure 13. Perturbation effects on phenotype changes in *S. cerevisiae* [15](#__RefHeading___Toc207085348)

Supplementary Figure 14. Perturbation effects on phenotype changes in *S. oneidensis* [16](#__RefHeading___Toc207085349)

Supplementary Figure 15. Relative perturbation influences on global phenotype changes [17](#__RefHeading___Toc207085350)

Supplementary Figure 16. Multi-goal metabolic engineering website main page [18](#__RefHeading___Toc207085351)

Supplementary Figure 17. Multi-goal metabolic engineering website tutorial page [19](#__RefHeading___Toc207085352)

Supplementary Figure 18. Correlation coefficients between different meta-phenotypes [20](#__RefHeading___Toc207085353)

**List of Tables**

Supplementary Table 1. Models and applied environmental and genetic perturbations [21](#__RefHeading___Toc207085354)

Supplementary Table 2. Engineering metric and goal definitions [22](#__RefHeading___Toc207085355)

Supplementary Table 3. Selected engineering designs with high total profit rates [23](#__RefHeading___Toc207085356)

Supplementary Table 4. Tabulated version of Figure 2 [24](#__RefHeading___Toc207085357)

Supplementary Table 5. Additional selected engineering designs [25](#__RefHeading___Toc207085358)

| A  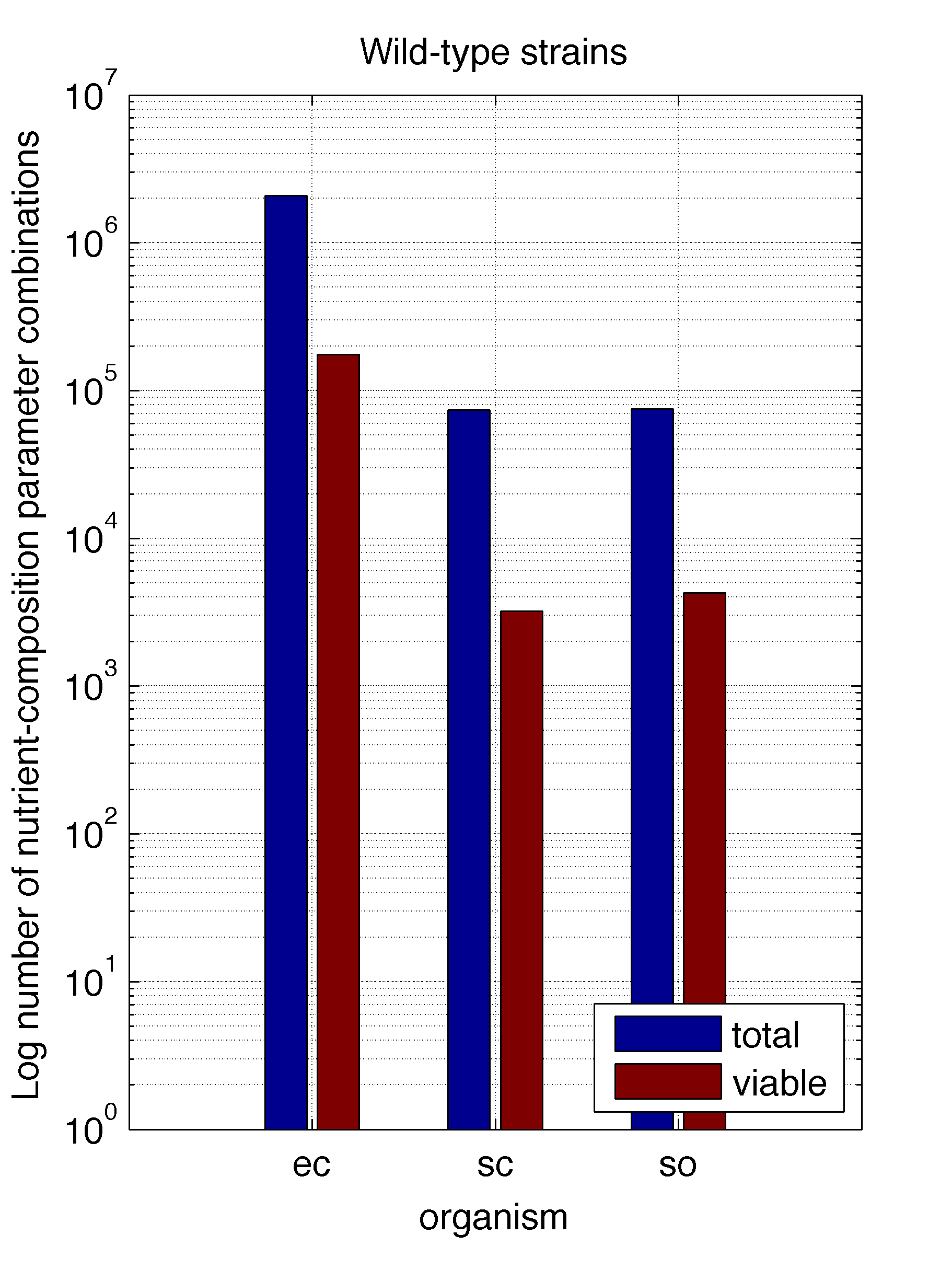 | B  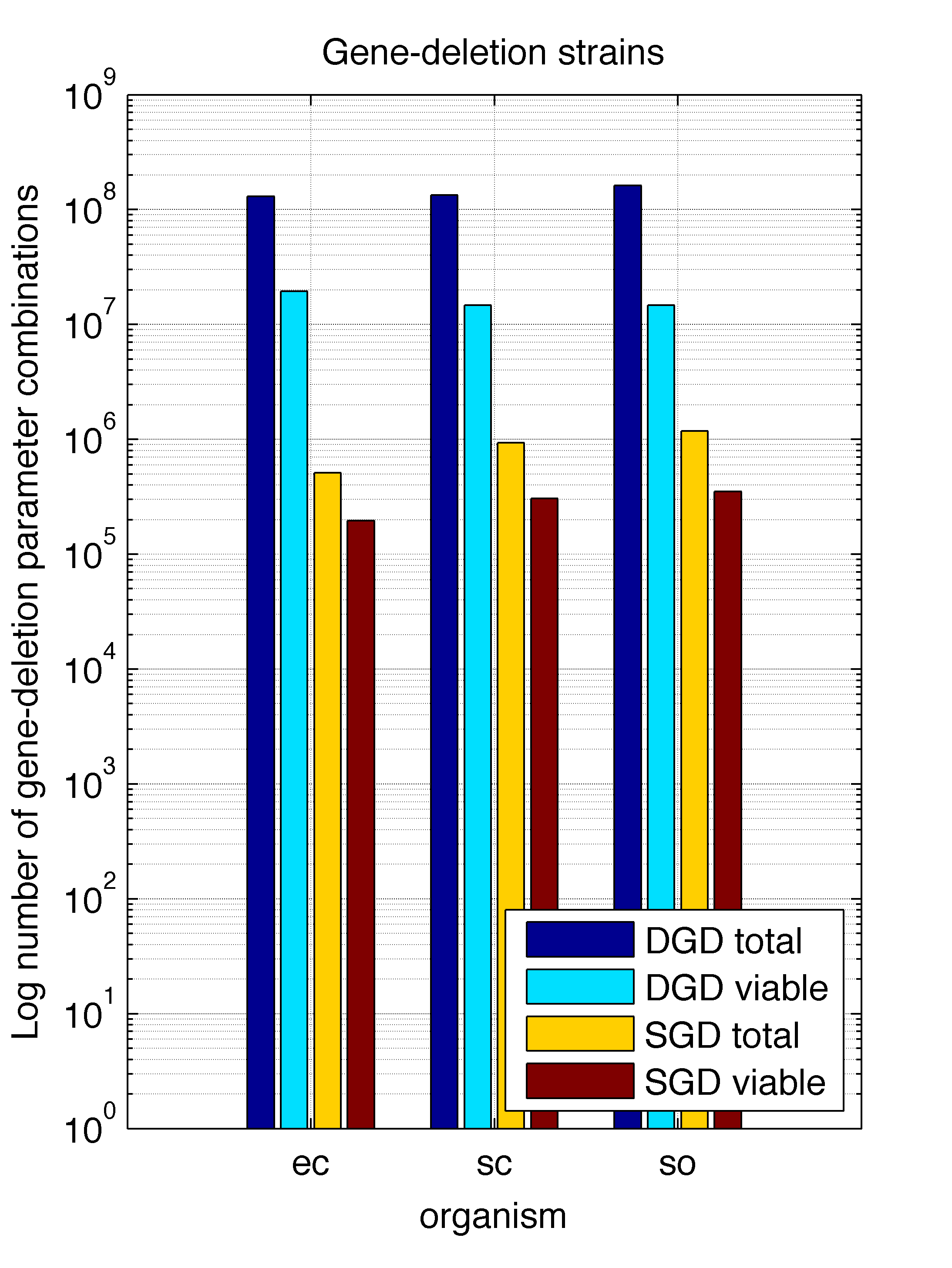 | C   |  | ec | sc | so | | --- | --- | --- | --- | | %  WT Viable | 8.37 | 5.78 | 4.30 | | %  SGD Viable | 38.33 | 24.80 | 39.64 | | % DGD Viable | 14.81 | 8.30 | 12.05 | |
| --- | --- | --- | --- | --- | --- | --- | --- | --- | --- | --- | --- | --- | --- | --- | --- | --- | --- | --- |

Supplementary Figure 1. Viable simulated conditions

(A) The number of total and viable wild-type nutrient-composition combinations. (B) The number of total and viable single and double gene-deletion combinations, which accounts for all viable wild-type nutrient-compositions applied to each gene-deletion mutant. (C) The percentage of viable-growth parameter combinations. Abbreviations: ec = *Escherichia coli*, sc = *Saccharomyces cerevisiae*, so = *Shewanella oneidensis*, WT = wild-type, SGD = single gene-deletions, and DGD = double gene-deletions.

| 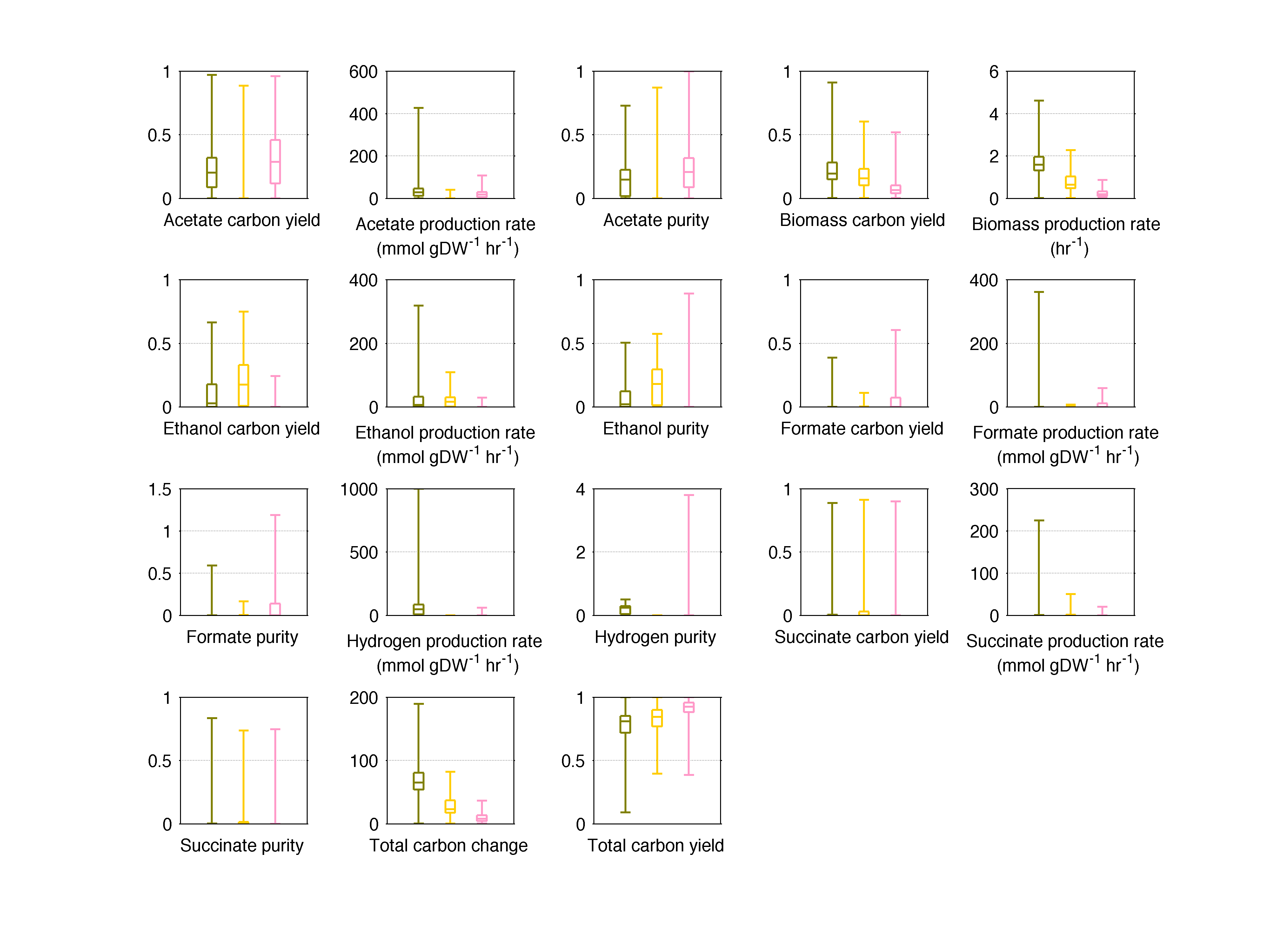 |
| --- |

Supplementary Figure 2. Box-plots of viable simulation results for complete data set

Vertical bars represent box-plot ranges for the complete simulation data set. In each subplot, the first box-plot is for *E. coli* (colored green), the second box-plot is for *S. cerevisiae* (colored orange), and the third box-plot is for *S. oneidensis* (colored pink).

| A  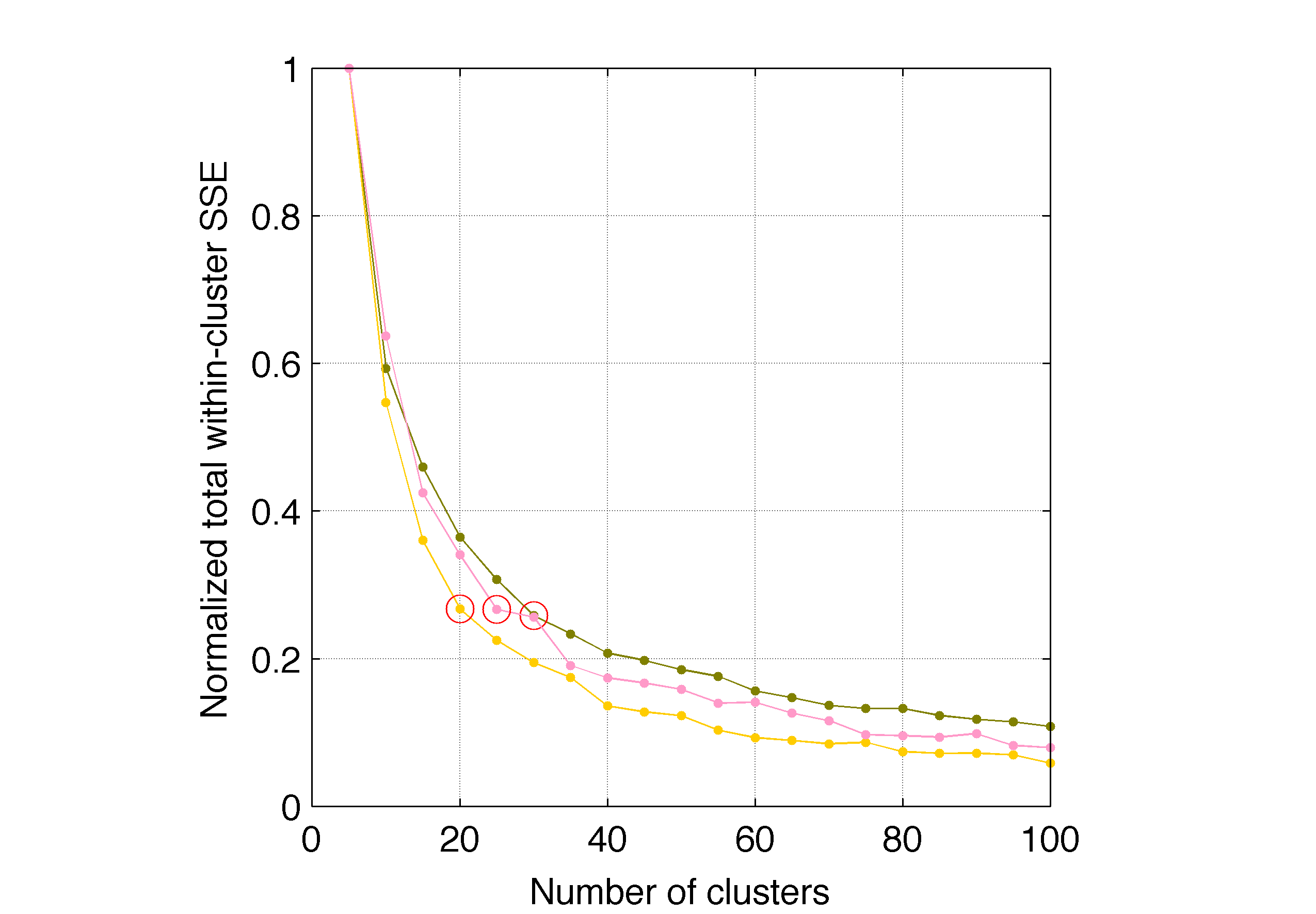 | B  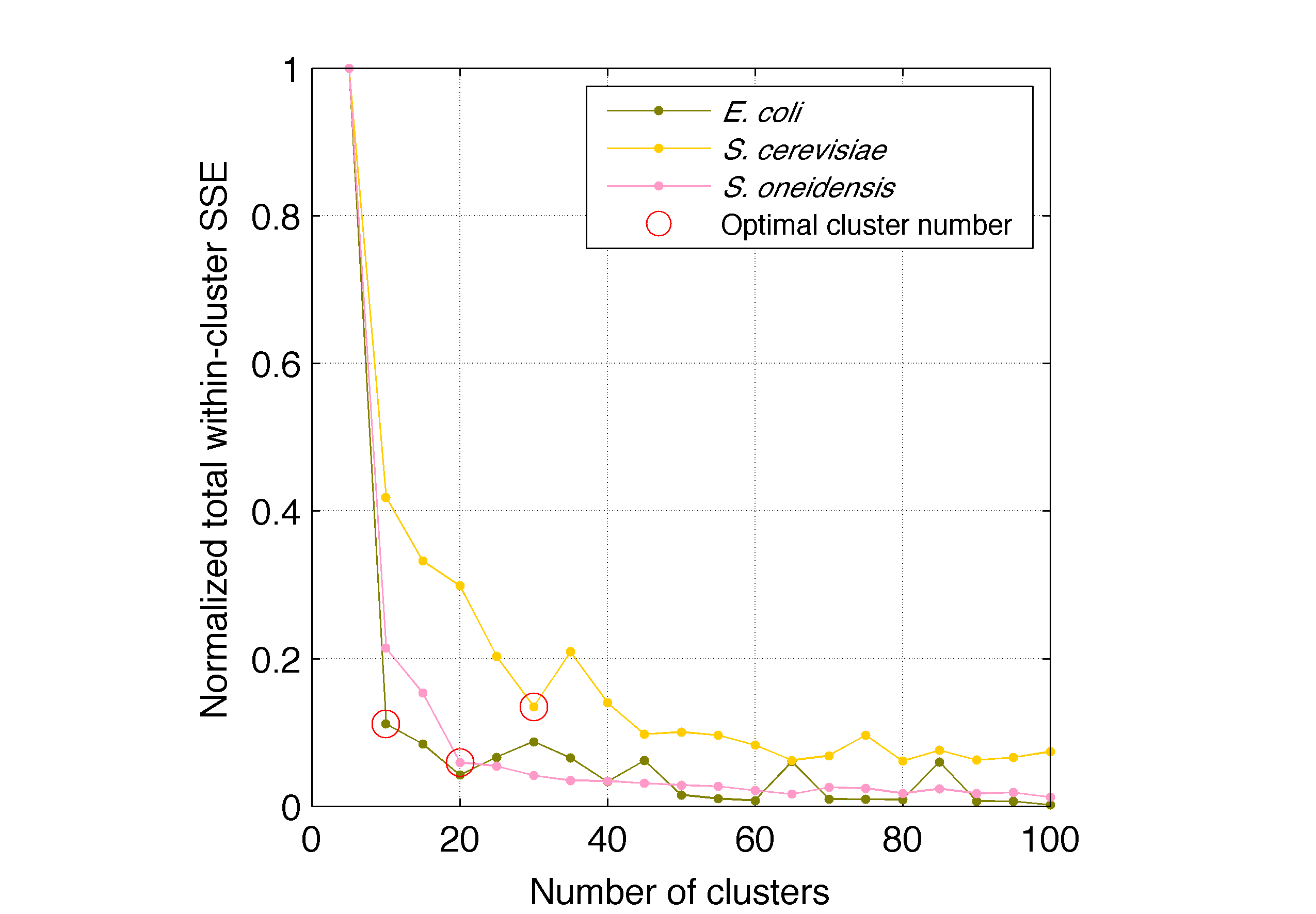 |
| --- | --- |

Supplementary Figure 3. Optimal phenotype cluster numbers

Optimal phenotype cluster numbers based on within-cluster sum of squared errors (SSE) for each phenotype cluster normalized by the maximum for each organism are shown for (A) the complete data set, and (B) the economic data subset. The markers for (A) and (B) are described in the legend shown in (B).

| 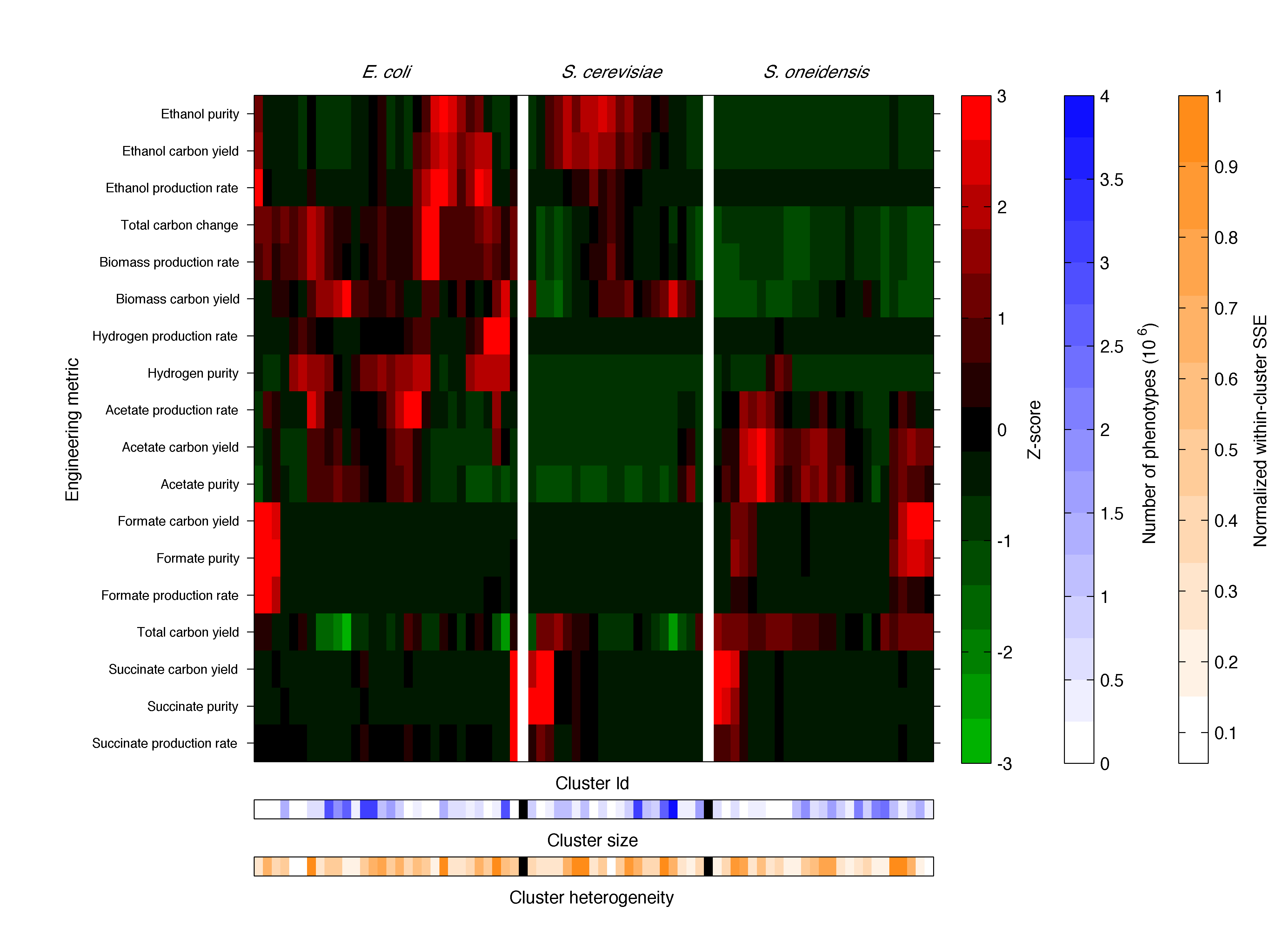 |
| --- |

Supplementary Figure 4. Engineering phenotype clusters for complete data set

Each column (listed by Cluster Id and grouped by organism) is the centroid associated with a corresponding k-means phenotype cluster for the complete simulation dataset without economic metrics. Cluster Ids increase sequentially, from left to right: 1 to 30 for *E. coli*, 31 to 50 for *S. cerevisiae*, and 51 to 75 for *S. oneidensis*. To compare across phenotype clusters and organisms, the metric values have been transformed into row-wise z-scores. All the rows and organism-specific columns were then hierarchically clustered. The “cluster sizes” are the number of individual phenotype simulations associated to each phenotype cluster. The “cluster heterogeneity” is the within-cluster sum of squared errors (SSE) for each phenotype cluster normalized by the maximum for each organism.

| 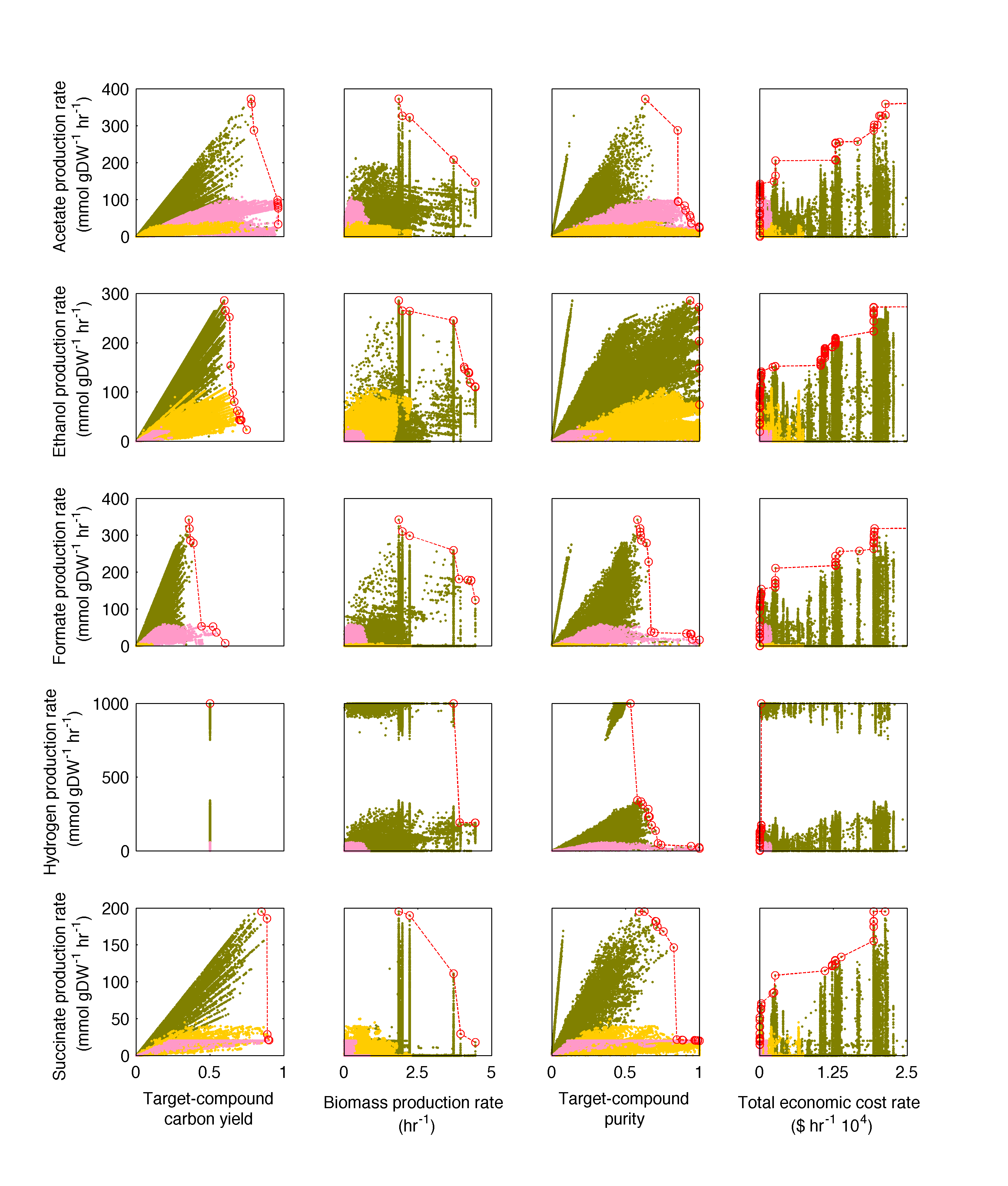 |
| --- |

Supplementary Figure 5. Pareto optima for target-compound production rates

Two-dimensional candidate designs and Pareto optimal designs and frontiers for target-compound production rates versus selected engineering goals. Markers are described in the legend shown in Supplementary Figure 8.

| A  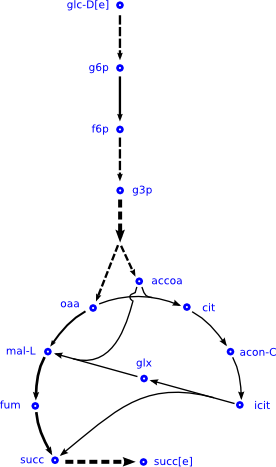 | B  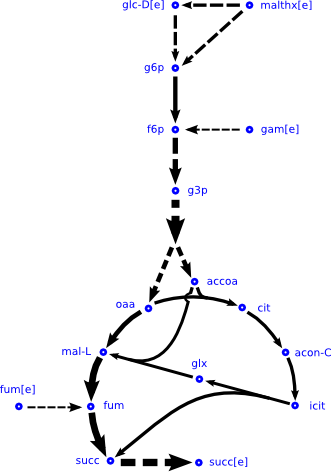 |
| --- | --- |

Supplementary Figure 6. Maximum theoretical succinate production in *E. coli*

Simplified metabolic network showing maximum theoretical succinate synthesis in *E. coli* using (A) glucose-D fermentation and (B) maltohexoase, fumarate and D-glucosamine anaerobic respiration. Nodes represent metabolites and edges represent reactions. A subset of reactions related to the glycolysis and citric acid cycle pathways are shown. Dashed edges represent consolidated multiple reactions. Edge thickness is approximately proportional to reaction flux. Metabolite abbreviations: accoa = acetyl-CoA, acon-C = cis-aconitate, cit = citrate, f6p = D-fructose 6-phosphate, fum = fumarate, fum[e] = extracellular fumarate, g3p = glyceraldehyde 3-phosphate, g6p = D-glucose 6-phosphate, gam[e] = extracellular D-glucosamine, glc-D[e] = extracellular D-glucose, glx = glyoxylate, icit = isocitrate, mal-L = L-malate, malthx[e] = extracellular maltohexoase, oaa = oxaloacetate, succ = succinate, succ[e] = extracellular succinate.

| A  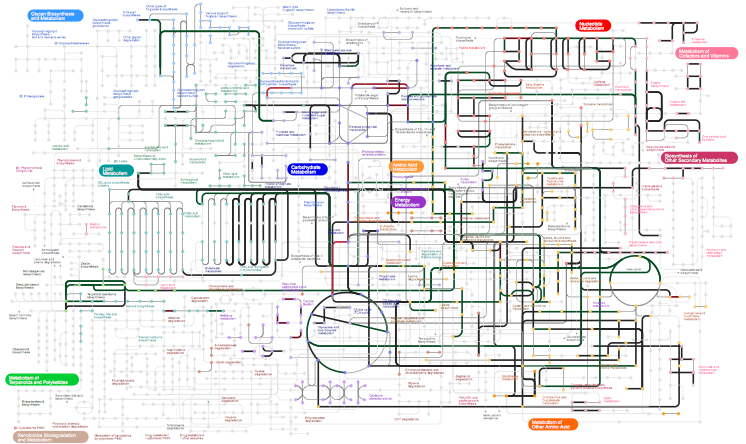 | C  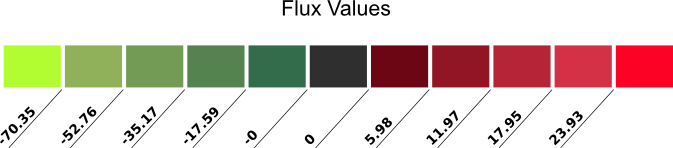  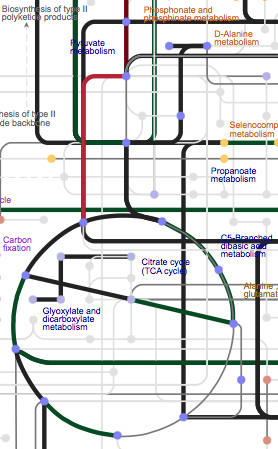 |
| --- | --- |
| B  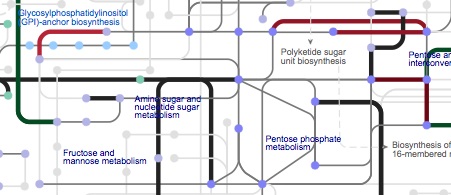 |

Supplementary Figure 7. Metabolic network pathway activity in *S. cerevisiae*

(A-C). Differential pathway activity between the wild-type *S. cerevisiae* grown aerobically on glucose minimal media and the mutant *S. cerevisiae* ΔYBR196C ΔYMR256C grown aerobically on glucose minimal media with urea as a nitrogen source (Design 5 in Table 1). (A) The complete metabolic network map, and close-ups (regions encircled by red boxes) of (B) carbohydrate metabolism and (C) the citric acid cycle. Metabolic pathway reactions are color-coded according to the relative flux differences (color legend for flux values in (C) applies to (A-C)) between the engineering designs. Similar metabolic network maps for all organisms and Pareto optimal designs can be viewed using the Multi-Goal Metabolic Engineering Website (Supplementary Figures 16 and 17).

| 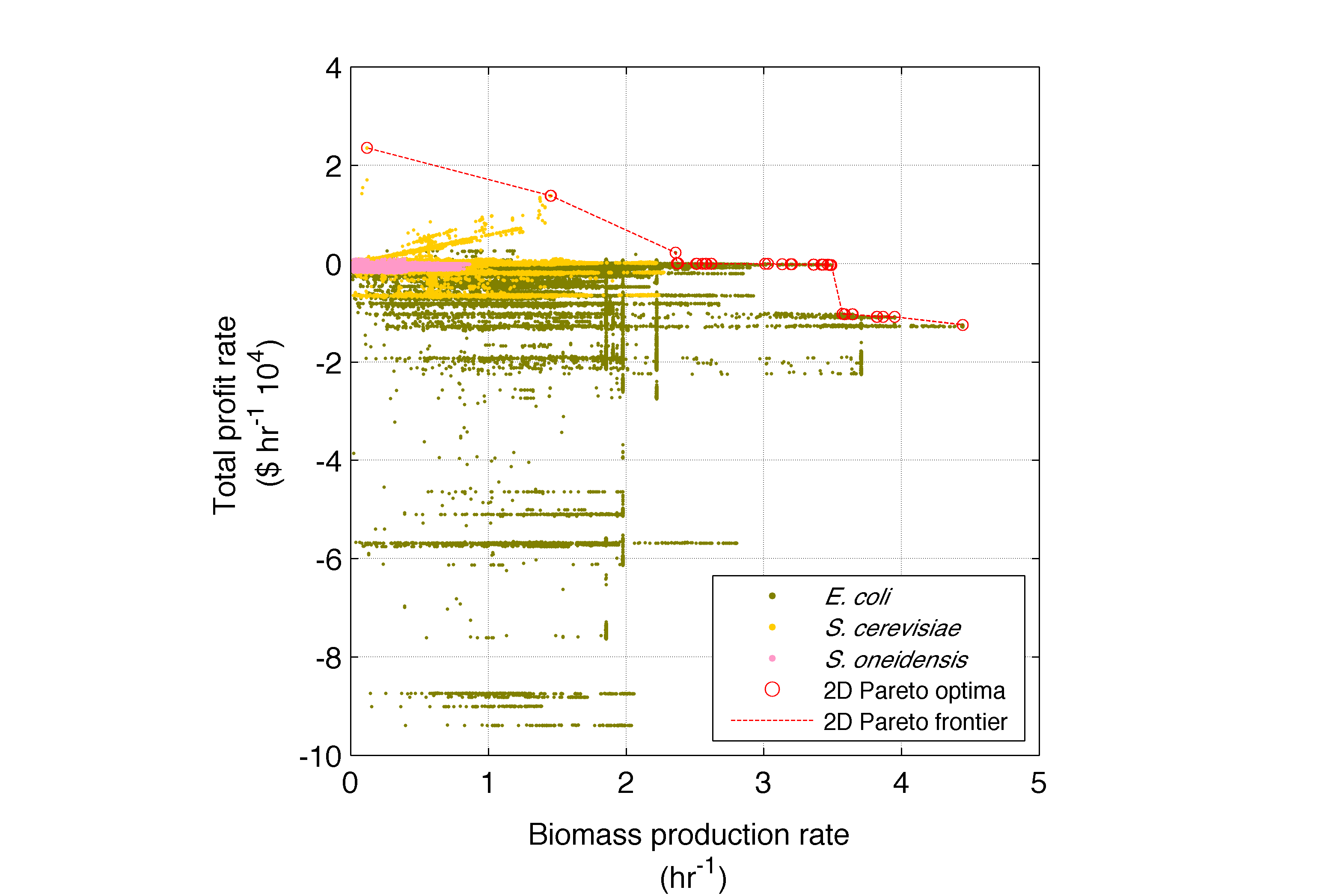 |
| --- |

Supplementary Figure 8. Pareto optima for total profit rate

Two-dimensional candidate designs and Pareto optimal designs and frontiers for total profit rate versus biomass production rate.

| 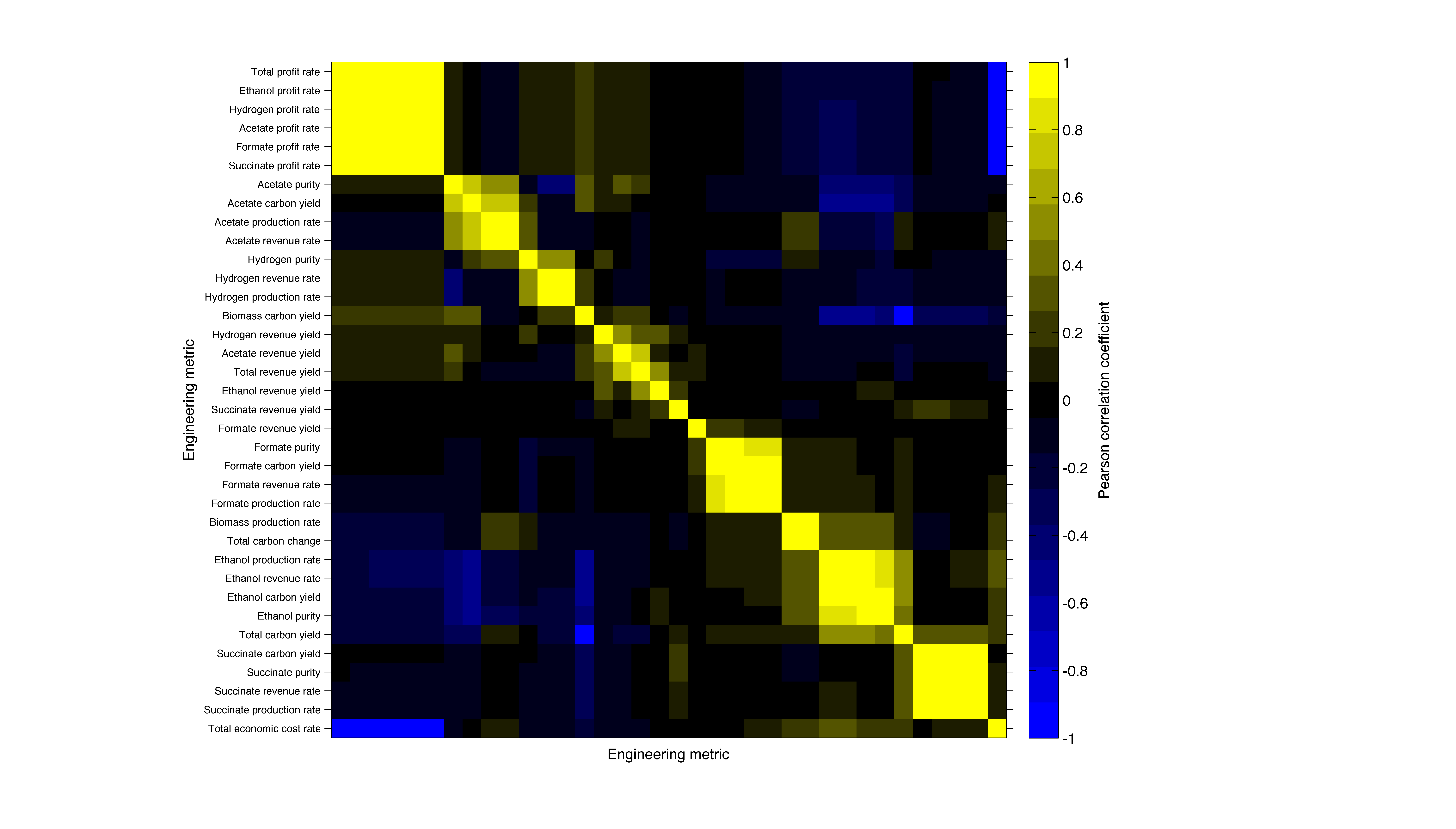 |
| --- |

Supplementary Figure 9. Pair-wise correlations of engineering metrics for *E. coli*

Pearson correlations computed between pairs of engineering metrics. Rows and columns are hierarchically clustered. Column labels are in the same order as the row labels, with the top metric row associated with the leftmost metric column.

| 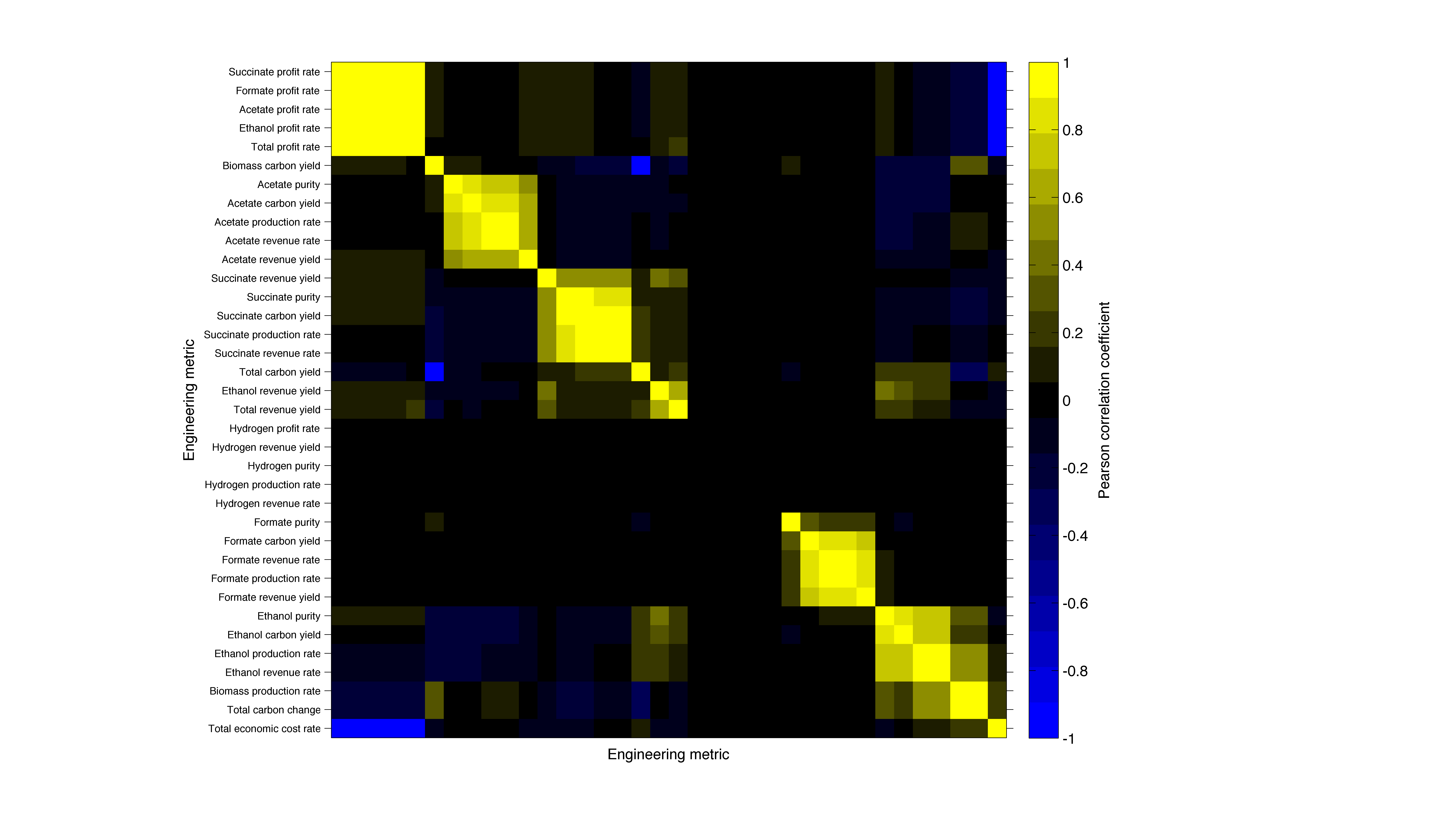 |
| --- |

Supplementary Figure 10. Pair-wise correlations of engineering metrics for *S. cerevisiae*

Pearson correlations computed between pairs of engineering metrics. Rows and columns are hierarchically clustered. Column labels are in the same order as the row labels, with the top metric row associated with the leftmost metric column.

| 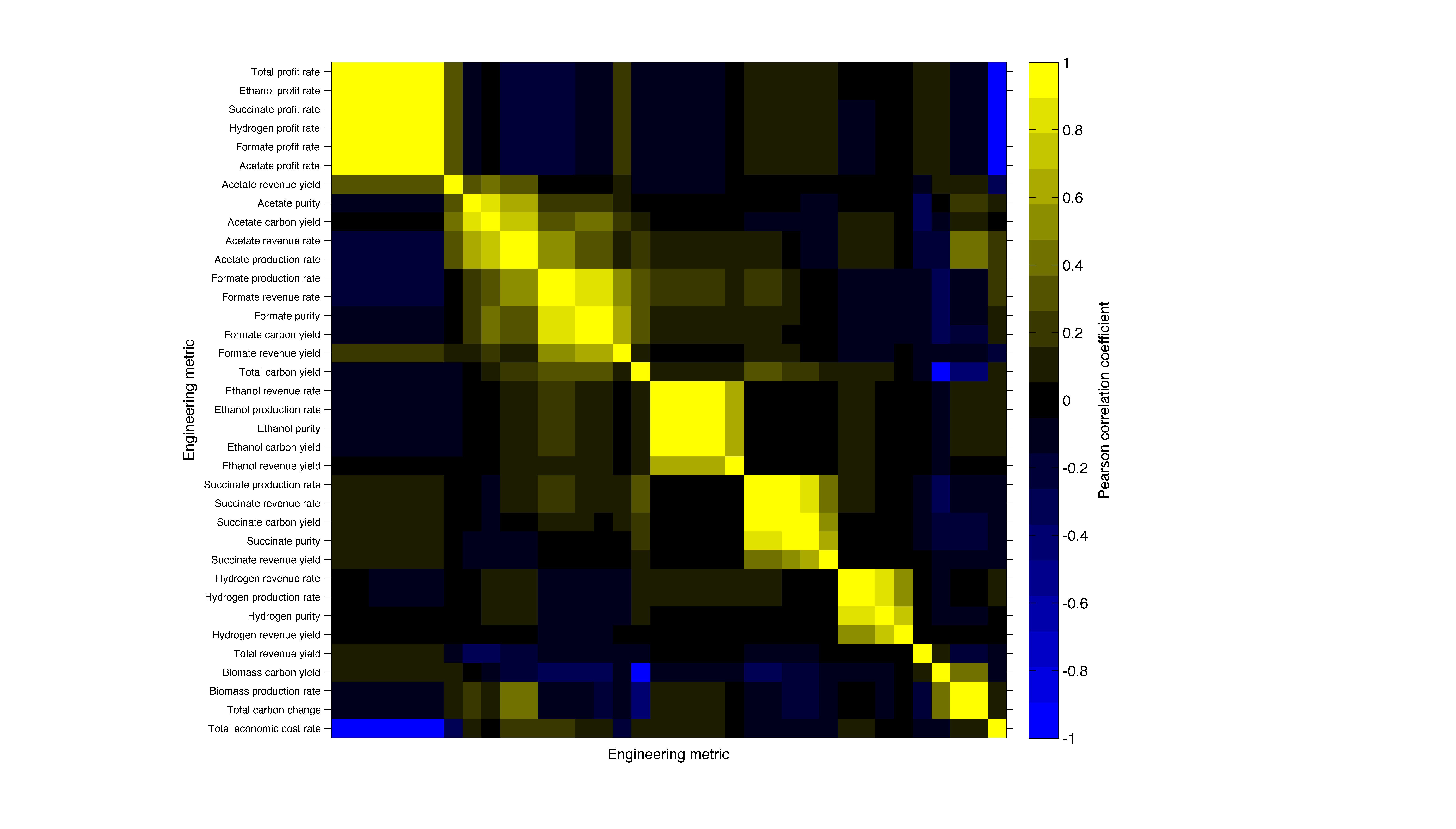 |
| --- |

Supplementary Figure 11. Pair-wise correlations of engineering metrics for *S. oneidensis*

Pearson correlations computed between pairs of engineering metrics. Rows and columns are hierarchically clustered. Column labels are in the same order as the row labels, with the top metric row associated with the leftmost metric column.

| 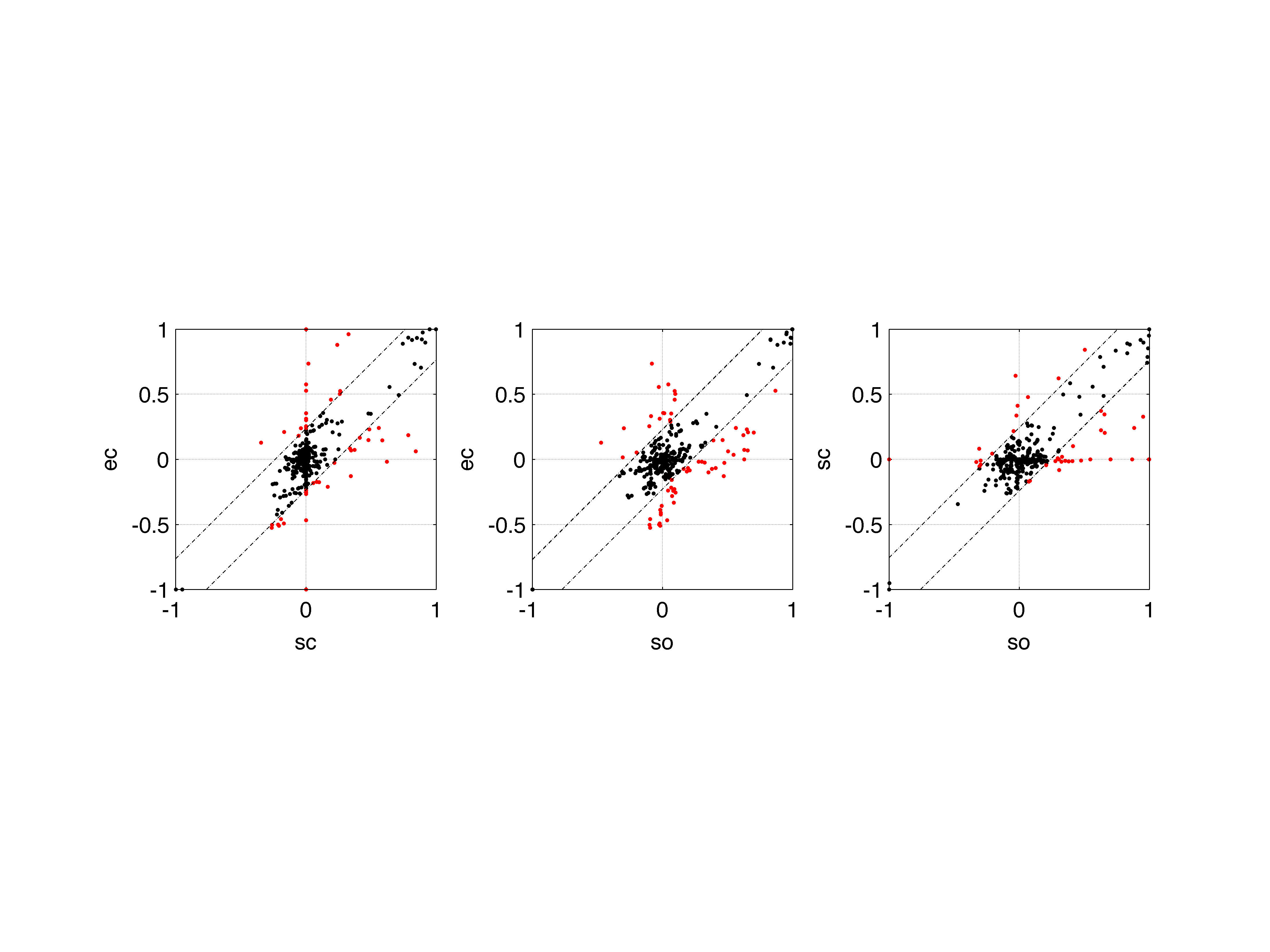 |
| --- |

Supplementary Figure 12. Pair-wise metric correlation comparisons between organisms

Dots indicate pair-wise metric Pearson correlation differences between organisms. Red dots are those correlations with absolute differences greater than two standard deviations (dashed lines) from the mean. Abbreviations: ec = *Escherichia coli*, sc = *Saccharomyces cerevisiae*, so = *Shewanella oneidensis*.

| A  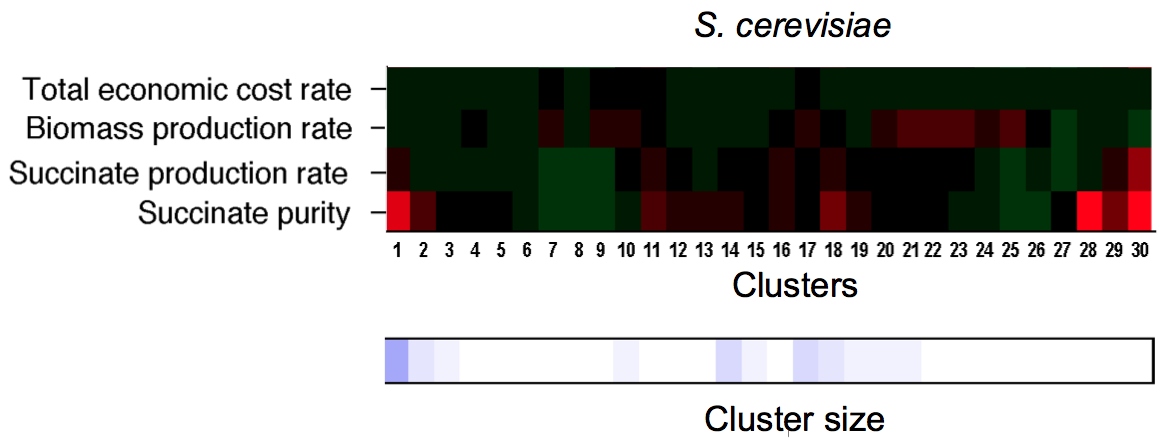 | | B  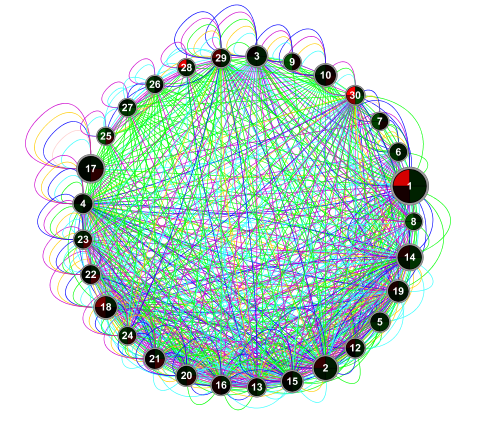 |
| --- | --- | --- |
| C  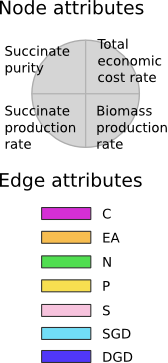 | 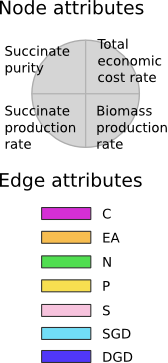 |

Supplementary Figure 13. Perturbation effects on phenotype changes in *S. cerevisiae*

(A) A subset of the engineering metrics associated with *S. cerevisiae* phenotype clusters (meta-phenotypes) shown in Figure 2. For values of engineering metrics (z-scores) and cluster sizes, refer to legend in Figure 2. (B) Meta-phenotype transition network for *S. cerevisiae*. Nodes *i* and *j* represent two viable-growth engineering meta-phenotypes (the nonviable-growth meta-phenotype is not shown). Node labels correspond to Clusters shown in (A). Node sizes are proportional to cluster sizes shown in (A). Edge *t_i,j_* represents the cumulative phenotype-cluster transition frequency between Nodes *i* and *j* due to a specified perturbation type. Edges are bidirectional, so *t_i,j_* is equivalent to *t_j,i_*. Edge thickness is proportional to the cumulative transition frequency for environmental or genetic perturbations. (C) Legend for meta-phenotype transition network in (B). Node faces are divided into quadrants that correspond to the selected engineering metrics shown in (A). Quadrant colors indicate the associated metric z-scores for the corresponding Cluster. Perturbation type (edge attribute) abbreviations: C = carbon sources, EA = electron acceptor sources, N = nitrogen sources, P = phosphorous sources, S = sulfur sources, SGD = single gene deletions, and DGD = double gene deletions.

| A  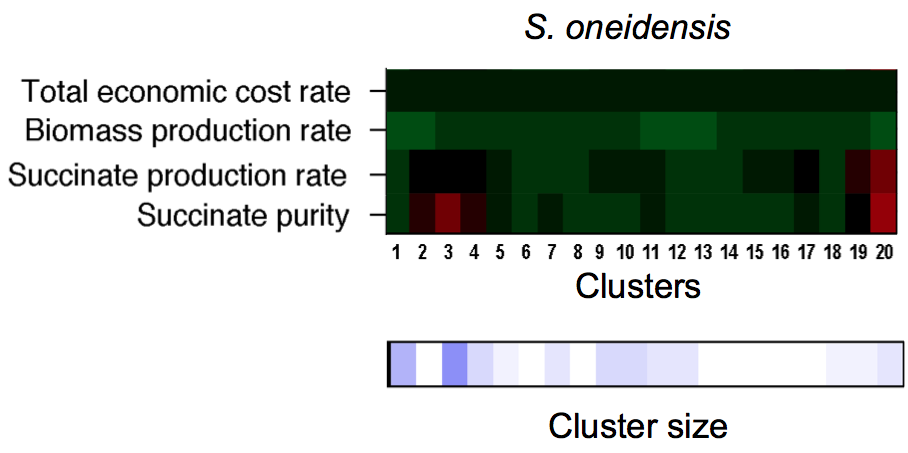 | | B  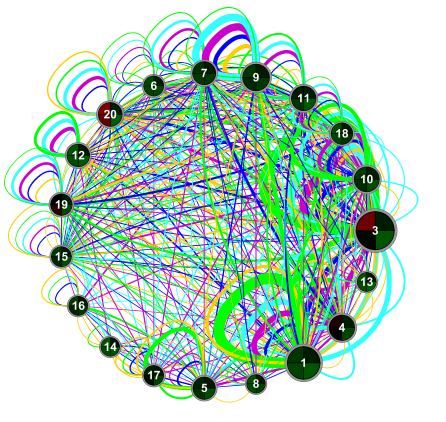 |
| --- | --- | --- |
| C  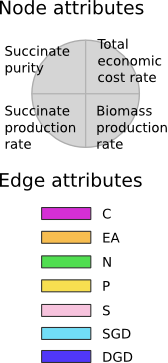 | 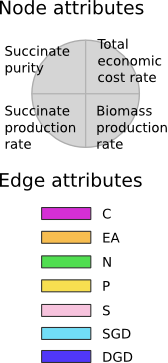 |

Supplementary Figure 14. Perturbation effects on phenotype changes in *S. oneidensis*

(A) A subset of the engineering metrics associated with *S. oneidensis* phenotype clusters (meta-phenotypes) shown in Figure 2. For values of engineering metrics (z-scores) and cluster sizes, refer to legend in Figure 2. (B) Meta-phenotype transition network for *S. oneidensis*. Nodes *i* and *j* represent two viable-growth engineering meta-phenotypes (the nonviable-growth meta-phenotype is not shown). Node labels correspond to Clusters shown in (A). Node sizes are proportional to cluster size shown in (A). Edge *t_i,j_* represents the cumulative phenotype-cluster transition frequency between Nodes *i* and *j* due to a specified perturbation type. Edges are bidirectional, so *t_i,j_* is equivalent to *t_j,i_*. Edge thickness is proportional to the cumulative transition frequency for environmental or genetic perturbations. (C) Legend for meta-phenotype transition network in (B). Node faces are divided into quadrants that correspond to the selected engineering metrics shown in (A). Quadrant colors indicate the associated metric z-scores for the corresponding Cluster. Perturbation type (edge attribute) abbreviations: C = carbon sources, EA = electron acceptor sources, N = nitrogen sources, P = phosphorous sources, S = sulfur sources, SGD = single gene deletions, and DGD = double gene deletions.

| 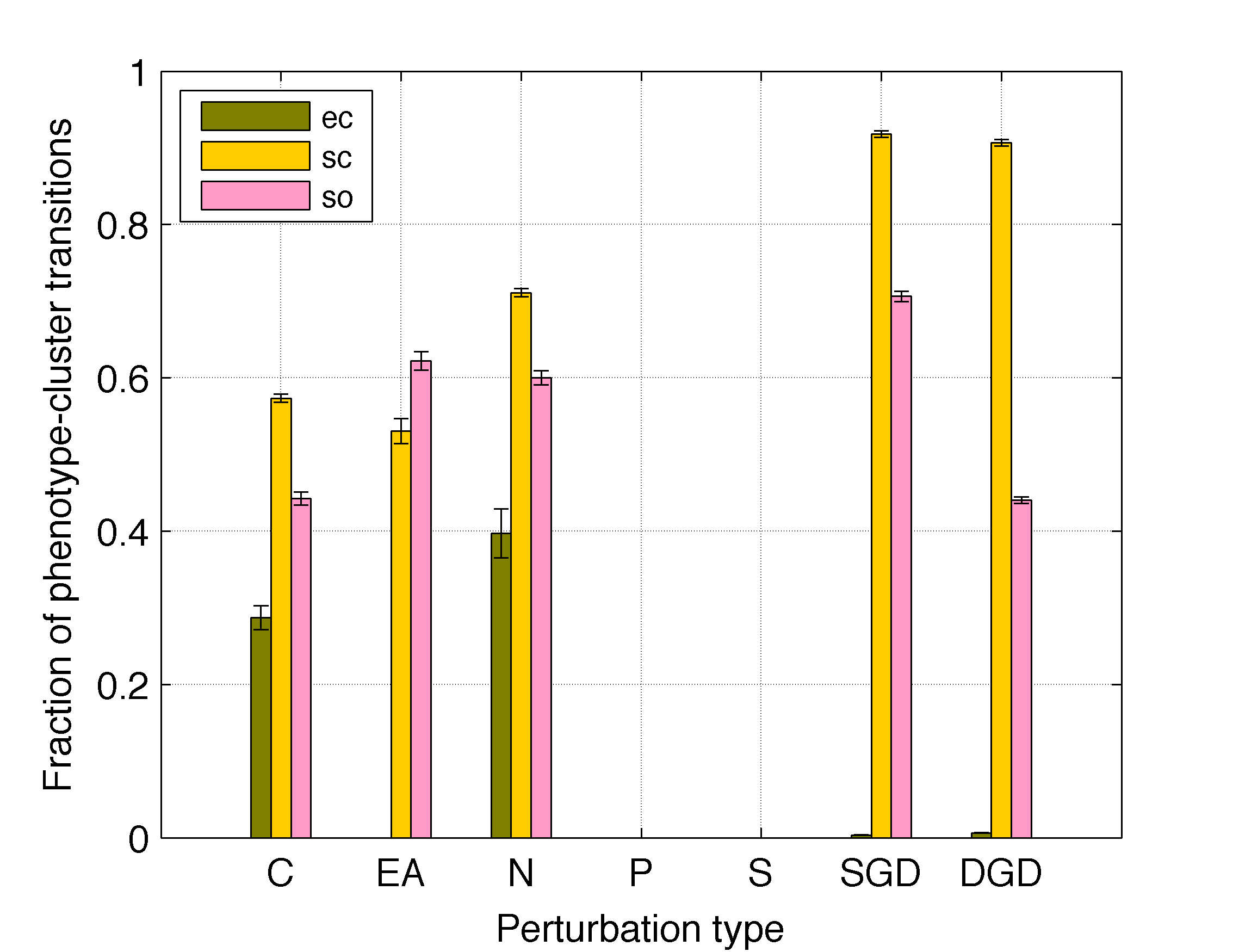 |
| --- |

Supplementary Figure 15. Relative perturbation influences on global phenotype changes

The total fraction and standard deviation for all organisms of each type of perturbation that causes meta-phenotype transitions. Each perturbation type was evaluated independently of other types. Abbreviations: ec = *Escherichia coli*, sc = *Saccharomyces cerevisiae*, so = *Shewanella oneidensis*, C = carbon sources, EA = electron acceptor sources, N = nitrogen sources, P = phosphorous sources, S = sulfur sources, SGD = single gene deletions, and DGD = double gene deletions.

| 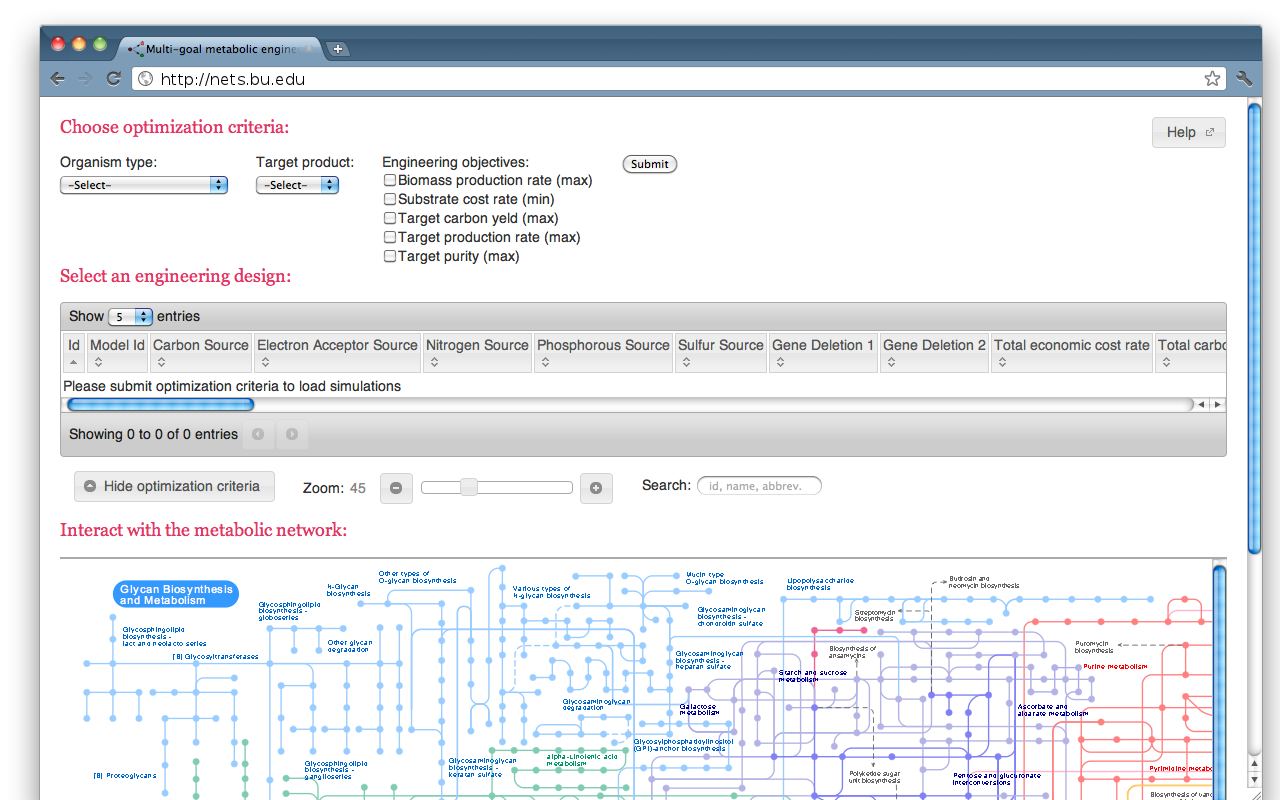 |
| --- |

Supplementary Figure 16. Multi-goal metabolic engineering website main page

The Multi-Goal Metabolic Engineering supplementary website’s main page is located at http://nets.bu.edu.

| 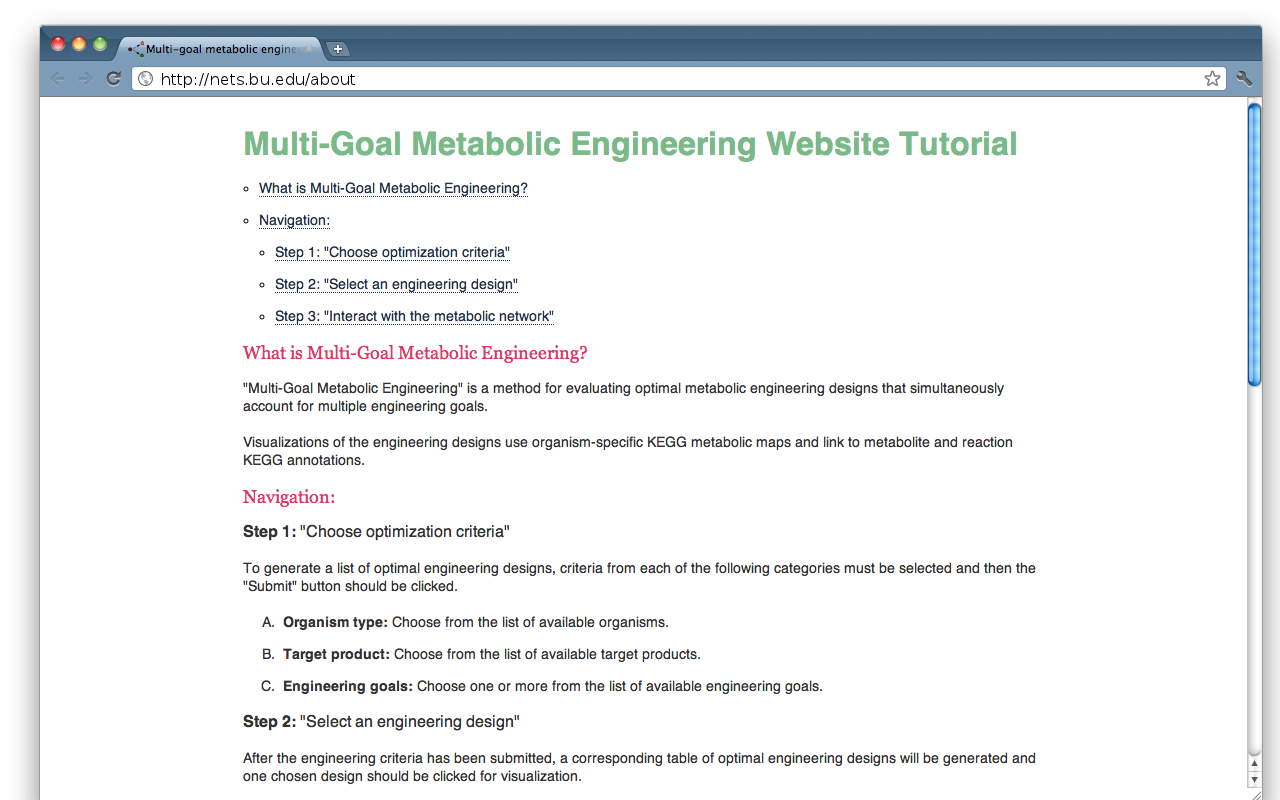 |
| --- |

Supplementary Figure 17. Multi-goal metabolic engineering website tutorial page

A tutorial for the website is located at http://nets.bu.edu/about. Alternatively the tutorial can be obtained by clicking “Help” on the website’s main page.

Supplementary Figure 18. Correlation coefficients between different meta-phenotypes

The correlation coefficient was computed for every pair of meta-phenotypes Z-score vectors (columns in Fig. 2, and Supplementary Table 4). The order of the meta-phenotypes (and their number) is the same as in Fig. 2 and Table 4. A notable feature of the ensuing matrix is the block structure, which reveals much higher similarity within than between organisms.

Supplementary Table 1. Models and applied environmental and genetic perturbations

The total number of parameter combinations evaluated was 133,420,920 for *Escherichia coli*, 179,133,985 for *Saccharomyces cerevisiae*, and 123,124,374 for *Shewanella oneidensis*.

|  |  | *Escherichia*  *coli* | *Saccharomyces cerevisiae* | *Shewanella oneidensis* |
| --- | --- | --- | --- | --- |
| Organism  model components | Model Id | iAF1260 | iMM904 | iSO783 |
|  | Literature reference | [1] | [2] | [3] |
|  | Genes | 1,260 | 904 | 783 |
|  | Metabolites | 1,039 | 1,228 | 634 |
|  | Reactions | 2,077 | 1,412 | 774 |
| Environmental perturbation types | Carbon nutrients | 106 | 76 | 19 |
|  | Electron acceptor nutrients | 7 | 1 | 15 |
|  | Nitrogen nutrients | 162 | 79 | 38 |
|  | Phosphorous nutrients | 2 | 1 | 1 |
|  | Sulfur nutrients | 4 | 2 | 2 |
| Genetic perturbation types | Single gene deletions | 512 | 289 | 276 |
|  | Double gene deletions | 130,816 | 41,616 | 37,950 |

Supplementary Table 2. Engineering metric and goal definitions

| Engineering metric | Mathematical definition | Explanation | Engineering goal |
| --- | --- | --- | --- |
| Biomass carbon yield | $\frac{c_{\text{carbon}}v_{\text{biomass}}}{\sum_{m} c_{\text{carbon}}v_{\text{feedstock}}}$ | Efficiency of the amount of carbon consumed from the feedstock and utilized for the production of biomass | Maximize |
| Biomass production rate (1/hr) | $v_{\text{biomass}}$ | Rate of biomass production | Maximize |
| Target-compound carbon yield | $\frac{c_{\text{carbon}}\text{v}_{\text{target}}}{\sum_{m} c_{\text{carbon}}v_{\text{feedstock}}}$ | Efficiency of the amount of carbon consumed from the feedstock and utilized for the production of a target-compound | Maximize |
| Target-compound production rate (mmol/gDW/hr) | $v_{\text{target}}$ | Rate of target-compound production | Maximize |
| Target-compound profit rate  ($/hr) |  | Rate of economic profit generated by the production of a target-compound | Maximize |
| Target-compound purity | $\frac{\text{v}_{\text{target}}}{\sum_{n} \text{v}_{\text{by-product}}}$ | Proportion of a target-compound produced relative to all non-biomass by-products. Low solubility gases such as carbon dioxide are not included. | Maximize |
| Target-compound revenue rate ($/hr) |  | Rate of economic revenue generated by the production of a target-compound | Maximize |
| Target-compound revenue yield | $\frac{c_{\text{price}}v_{\text{target}}}{\sum_{m} c_{\text{price}}v_{\text{feedstock}}}$ | Efficiency of the economic revenue generated by producing a target-compound relative to the total economic cost | Maximize |
| Total carbon change  (mmol/gDW/hr) | $\sum_{m} c_{\text{carbon}}v_{\text{feedstock}}-\sum_{n} c_{\text{carbon}}v_{\text{by-products}}$ | Net change of the amount of carbon consumed from the feedstock minus the amount of carbon synthesized into non-biomass by-products | Maximize |
| Total carbon yield | $\frac{\sum_{n} c_{\text{carbon}}v_{\text{by-products}}}{\sum_{m} c_{\text{carbon}}v_{\text{feedstock}}}$ | Efficiency of the amount of carbon consumed from the feedstock to that synthesized into non-biomass by-products | Maximize |
| Total economic cost rate  ($/hr) |  | Rate of economic cost for the consumed feedstock | Minimize |
| Total profit rate ($/hr) |  | Total economic profit rate generated by recovering the total non-biomass by-product revenue minus the total economic cost | Maximize |
| Total revenue yield | $\frac{\sum_{n} c_{\text{price}}v_{\text{by-product}}}{\sum_{m} c_{\text{price}}v_{\text{feedstock}}}$ | Proportion of the economic revenue generated by recovering the total value of the non-biomass by-products relative to the total economic cost | Maximize |

Note: *m* refers to the overall number of feedstock uptake reactions, whereas *n* refers to the overall number of by-product secretion reactions.

Supplementary Table 3. Selected engineering designs with high total profit rates

| Organism ^a^ | Gene  deletions | Nutrient  sources ^b^ | High-value  byproducts ^c^ | Total economic  cost rate  ($ hr^-1^) | Total profit  Rate  ($ hr^-1^) |
| --- | --- | --- | --- | --- | --- |
| sc | YLL052C, YPR192W | pacald, NA, adn, pi, so4 | pap (0.99) | 16.9031 | 23579.9 |
| sc | YCR012W, YDR050C | sbt-D, o2, trp-L, pi, so4 | nadp (1.00) | 4.49168 | 15511.6 |
| sc | YJL121C, YNL241C | sucr, o2, asn-L, pi, so4 | lanost (0.99) | 7.56811 | 13823 |
| ec | purT, lpd | cit, no3, gthrd, pi, so4 | cgly (1.00) | 41.7945 | 2607.38 |
| so | SO0970, SO2916 | akg, o2, ser-L, pi, so4 | glyc-R (0.91) | 10.2442 | 1010.79 |

^a^ Organism abbreviations: ec = *Escherichia coli*, sc = *Saccharomyces cerevisiae,* so = *Shewanella oneidensis*. ^b^ Nutrients are ordered by nutrient type: carbon, electron acceptor, nitrogen, phosphorous, and sulfur source. “NA” indicates that no nutrient source of that type was provided. ^c^ “High value byproducts” are metabolites that are excreted and substantially contribute to the total profit rate. The proportion of the total revenue rate associated with each byproduct is shown in parenthesis. Metabolite abbreviations: adn = Adenosine, akg = 2-Oxoglutarate, asn-L = L-Asparagine, cgly = Cys-Gly, cit = Citrate, glyc-R = (R)-Glycerate, gthrd = Reduced glutathione, lanost = Lanosterol, nadp = Nicotinamide adenine dinucleotide phosphate, no3 = Nitrate, o2 = O2, pacald = Phenylacetaldehyde, pap = Adenosine 3',5'-bisphosphate, pi = Phosphate, sbt-D = D-Sorbitol, ser-L = L-Serine, so4 = Sulfate, sucr = Sucrose, trp-L = L-Tryptophan.

Supplementary Table 4. Tabulated version of Figure 2

Excel Spreadsheet “Supplementary_Table_4.xls”

Supplementary Table 5. Additional selected engineering designs (a comprehensive list of all phenotype metrics and associated conditions may downloaded or viewed using the online tool)

| **Design criteria** | | **Design annotations** | | | **Design metrics** | | | | |
| --- | --- | --- | --- | --- | --- | --- | --- | --- | --- |
| **Design goal ^a^** | **Design type ^b^** | **Gene deletions** | **Nutrient sources ^c^** | **Organism ^d^** | **Biomass production rate (hr^-1^)** | **Target carbon yield** | **Total economic cost rate ($ hr^-1^)** | **Target production rate (mmol gDW^-1^ hr^-1^)** | **Target purity** |
| Acetate production rate | candidate | pstS, potE | malthx, fum, acgam1p, ppt, so4 | ec | 1.85 | 0.78 | 76256 | 373.04 | 0 |
| Acetate carbon yield | candidate | SO4754, SO3440 | etoh, cobalt3, gly, pi, so4 | so | 0.05 | 0.96 | 52.64 | 33.32 | 0.62 |
| Ethanol production rate | candidate | cycA, b3469 | malthx, fum, acgam1p, NA, so4 | ec | 1.85 | 0.6 | 76224.6 | 285.99 | 0.5 |
| Ethanol carbon yield | candidate | YBR011C, YMR205C | acald, NA, gam6p, NA, so4 | sc | 0.07 | 0.75 | 535.49 | 22.82 | 0.54 |
| Formate production rate | candidate | glpC, mtr | malthx, fum, acgam1p, NA, so4 | ec | 1.85 | 0.36 | 76280.3 | 341.95 | 0.34 |
| Formate carbon yield | candidate | SO2913, SO0425 | ppa, fe3, ser-L, pi, so4 | so | 0.03 | 0.6 | 4.36 | 7.01 | 0.49 |
| Succinate production rate | microarray | gnd, uraA | malthx, fum, gam6p, pi, so4 | ec | 1.85 | 0.85 | 21296.7 | 195.34 | 0.26 |
| Succinate carbon yield | microarray | YJR077C, YOL126C | mal-L, NA, orn, pi, so4 | sc | 0.02 | 0.9 | 8.32 | 20.6 | 0.61 |

^a^ The specified engineering design goal is used to determine the optimal engineering designs for the corresponding “Design type”. ^b^ “Validated” designs are designs that were experimentally validated, “microarray” designs are designs that had matching experimental microarray data, and “candidate” designs are simulated designs that are neither “validated” nor “microarray” designs. ^c^ Nutrients are ordered by nutrient type: carbon, electron acceptor, nitrogen, phosphorous, and sulfur source. “NA” indicates that no nutrient source of that type was provided. Metabolite abbreviations: acald = Acetaldehyde, acgam1p = N-Acetyl-D-glucosamine 1-phosphate, cobalt3 = Co3+, etoh = Ethanol, fe3 = Fe3+, fum = Fumarate, gam6p = D-Glucosamine 6-phosphate, gly = Glycine, glu-L = L-Glutamate, mal-L = L-Malate, malthx = Maltohexoase, orn = Ornithine, pi = Phosphate, ppa = Propionate, ser-L = L-Serine, so4 = Sulfate. ^d^ Organism abbreviations: ec = *Escherichia coli*, sc = *Saccharomyces cerevisiae,* so = *Shewanella oneidensis*.

**References**

1. Feist AM, Henry CS, Reed JL, Krummenacker M, Joyce AR, Karp PD, Broadbelt LJ, Hatzimanikatis V, Palsson BØ: **A genome-scale metabolic reconstruction for Escherichia coli K-12 MG1655 that accounts for 1260 ORFs and thermodynamic information**. *Mol. Syst. Biol* 2007, **3**:121.

2. Mo ML, Palsson BO, Herrgård MJ: **Connecting extracellular metabolomic measurements to intracellular flux states in yeast**. *BMC Syst Biol* 2009, **3**:37.

3. Pinchuk GE, Hill EA, Geydebrekht OV, De Ingeniis J, Zhang X, Osterman A, Scott JH, Reed SB, Romine MF, Konopka AE, Beliaev AS, Fredrickson JK, Reed JL: **Constraint-based model of Shewanella oneidensis MR-1 metabolism: a tool for data analysis and hypothesis generation**. *PLoS Comput. Biol* 2010, **6**:e1000822.
